# Autologous skeletal muscle derived cells expressing a novel functional dystrophin provide a potential therapy for Duchenne Muscular Dystrophy

**DOI:** 10.1038/srep19750

**Published:** 2016-01-27

**Authors:** Jinhong Meng, John R. Counsell, Mojgan Reza, Steven H. Laval, Olivier Danos, Adrian Thrasher, Hanns Lochmüller, Francesco Muntoni, Jennifer E. Morgan

**Affiliations:** 1 The Dubowitz Neuromuscular Centre, Molecular Neurosciences Section, Developmental Neurosciences Programme, UCL Institute of Child Health, 30 Guilford Street, London, UK, WC1N 1EH; 2 UCL Cancer Institute, Paul O’Gorman Building, University College London, 72 Huntley Street, London, UK, WC1E 6BT; 3 John Walton Centre for Muscular Dystrophy Research, MRC Centre for Neuromuscular Diseases, Institute of Genetic Medicine, Newcastle University, Newcastle upon Tyne, UK, NE1 3BZ; 4 Molecular and Cellular Immunology, Institute of Child Health, University College London, 30 Guilford Street, London, UK, WC1N 1EH

## Abstract

Autologous stem cells that have been genetically modified to express dystrophin are a possible means of treating Duchenne Muscular Dystrophy (DMD). To maximize the therapeutic effect, dystrophin construct needs to contain as many functional motifs as possible, within the packaging capacity of the viral vector. Existing dystrophin constructs used for transduction of muscle stem cells do not contain the nNOS binding site, an important functional motif within the dystrophin gene. In this proof-of-concept study, using stem cells derived from skeletal muscle of a DMD patient (mdcs) transplanted into an immunodeficient mouse model of DMD, we report that two novel dystrophin constructs, C1 (ΔR3-R13) and C2 (ΔH2-R23), can be lentivirally transduced into mdcs and produce dystrophin. These dystrophin proteins were functional *in vivo*, as members of the dystrophin glycoprotein complex were restored in muscle fibres containing donor-derived dystrophin. In muscle fibres derived from cells that had been transduced with construct C1, the largest dystrophin construct packaged into a lentiviral system, nNOS was restored. The combination of autologous stem cells and a lentivirus expressing a novel dystrophin construct which optimally restores proteins of the dystrophin glycoprotein complex may have therapeutic application for all DMD patients, regardless of their dystrophin mutation.

Duchenne Muscular Dystrophy (DMD) is a severe inherited muscle disease that affects 1 in 5,000 newborn boys^1^. The lack of dystrophin^2^,^3^ leads to continuous cycles of degeneration and regeneration of muscle fibres and at the later stages of the disease, replacement of muscle by fat or fibrotic tissue, which greatly compromises muscle function.

Stem cell therapy is a promising strategy for the treatment of DMD, as transplanted stem cells fuse with existing muscle fibres and restore functional dystrophin expression at the sarcolemma, to prevent further progression of the disease. Stem cells isolated from human skeletal muscle^4^,^5^,^6^,^7^ have been shown to contribute to muscle regeneration after transplantation into animal models. Among these, human skeletal muscle derived pericytes^5^, or similar muscle derived cells (mdcs)^6^ and CD133+ cells^4^,^7^,^8^ are particularly effective. Transplantation of muscle stem cells isolated from a normal donor into patient muscle (allograft) will restore full length, functional dystrophin protein in regenerated muscle fibres, but this would elicit immune rejection. To avoid this, an *ex vivo* strategy can be implemented, involving autologous transplantation of the patient’s stem cells following genetic correction *in vitro*.

Lentiviruses are able to transduce non-dividing and dividing cells, leading to stable and long-term gene expression^9^, but they only have a small cloning capacity^10^ and therefore cannot accommodate the cDNA of full length dystrophin (>11 Kb). To circumvent this limitation, truncated forms of dystrophin have been developed, retaining dystrophin domains thought to serve essential functions^11^,^12^. However, not all of these truncated dystrophins are fully functional. Most dystrophin constructs that have been used in combination with experimental cell transplantation strategies lack the spectrin repeats 16 and 17 (R16–17), which is a well-recognized nNOS binding site^13^,^14^, thus would not exert the signaling function via the nNOS pathway. We therefore developed 2 novel mini-dystrophin constructs, a 4.2 Kb ΔH2–R23 (C2) construct which contained only the critical functional motifs of the dystrophin and ΔR3–R13 (C1), a large dystrophin construct near the size limit of the packaging capacity of the lentivirus. Importantly, C1 contains the nNOS binding site-spectrin repeats 16 and 17. These constructs were lentivirally-transduced into human muscle stem cells and their efficacy compared.

For clinical application, it is desirable that dystrophin expression occurs only in cells of the muscle lineage, so the use of viral promoters, such as cytomegalovirus (CMV) or spleen focus-forming virus (SFFV), that would drive gene expression in all cell types, would not be ideal. One muscle specific promoter, the human desmin (hDesmin) promoter offers a comparable level of transgene expression to CMV^15^ and SFFV promoters^16^. In light of this, we employed the hDesmin promoter in our investigations, with the additional advantage of restricting transgene expression to myogenic cells. However, the large size of the hDesmin promoter (1.8 Kb) presented a technical drawback. To circumvent this limitation and enable efficient packaging of larger dystrophin constructs, we also produced lentiviral vectors expressing dystrophin under the control of the much smaller SFFV promoter (412 bp).

We show that cells derived from skeletal muscle of a DMD patient^6^ can be transduced with lentiviruses expressing dystrophin constructs. Dystrophin protein was produced by these cells *in vitro* and in regenerated muscle fibres of donor origin within host *mdx* nude mouse muscles that had been grafted with donor cells. In the regenerated muscle fibres that expressed dystrophin, components of the dystrophin-associated glycoprotein complex (DGC) were restored in a dose-dependent manner. In fibres expressing dystrophin C1, nNOS was also present at the sarcolemma, suggesting that the C1 dystrophin construct is superior to C2 in that it can also restore the nNOS signaling pathway *in vivo*.

## Results

### Generation of dystrophin lentivirus and titration

**Figure 1:**
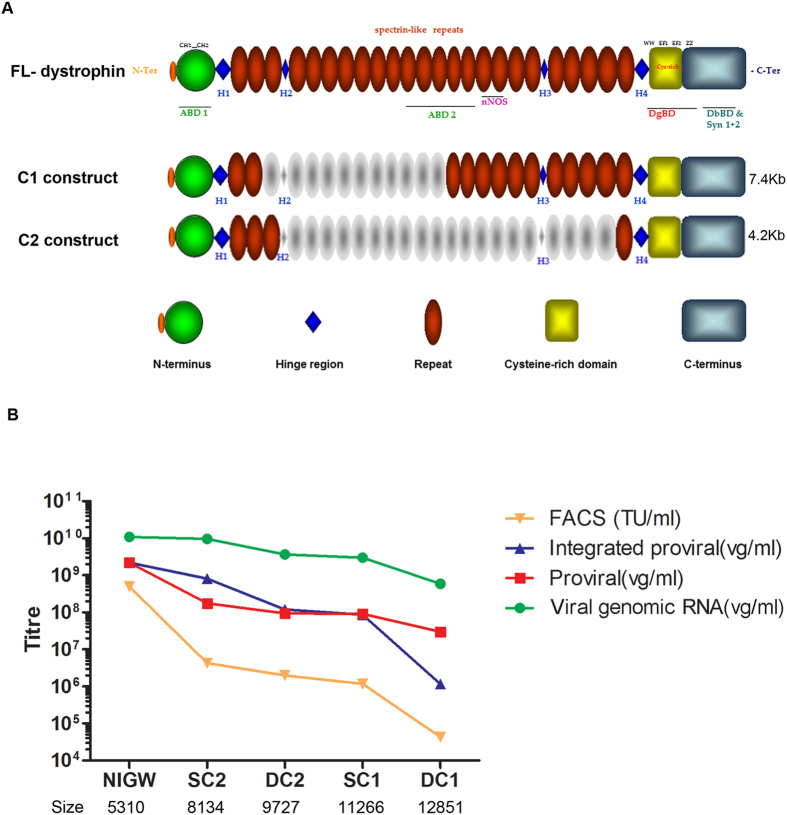
Schematic illustration of mini-dystrophin constructs and the titration of lentivirus using different methods. (**A**) Schematic illustration of mini-dystrophin constructs compared to the full-length dystrophin. The sizes of mini-dystrophin constructs are given on the right. Individual deletions are outlined in grey and domains highlighted in different colors as indicated. (**B**) Titration of lentivirus using different methods, showing that the titration of the lentiviruses was limited by the size (bp) of the inserted transgene. NIGW: pRRL.SFFV.neo-IRES-GFP; SC2: SFFV-C2-GFP; DC2: hDesmin-C2-GFP; SC1: SFFV-C1-GFP; DC1: hDesmin-C1-GFP.

The structure of the dystrophin constructs examined in our study is shown in Fig. 1A. The sizes (from 5′-3′UTR) of construct C1 and C2 are 7.4 Kb and 4.2 Kb respectively. After being cloned into the lentiviral vector, under different promoters, some of the lentiviral constructs constituted more than 10 Kb between the flanking LTRs (Fig. 1B), which is beyond the optimal range of lentivirus packaging capacity^10^,^17^. In order to assess the efficiency of viral delivery of large constructs, lentiviruses containing SFFV-C1-GFP, SFFV-C2-GFP, hDesmin-C1-GFP or hDesmin-C2-GFP were produced using a third-generation lentiviral packaging system^18^ and titred by a range of methods (Fig. 1B) and compared to a smaller pRRL.SFFV.neo-IRES-GFP-WPRE construct (NIGW, 5310 bp). Titration of the total viral RNA copy number and the transduced proviral copy number showed a trend towards reduced vector titre as the length of packaged sequence increased. However, Alu-PCR titration highlighted that lentiviral integration efficiency reduced at a greater rate as the length of packaged sequence exceeded 11.5 kb. This profile was confirmed by FACS titration, which highlighted a sharp drop in functional titre above the 11.5 kb level. Given that non-integrating vectors are reported to express with a lower intensity than integrated proviruses^19^, this reduced rate of integration may partly explain the rapid drop-off in FACS titre experienced with larger vectors.

### Dystrophins C1 and C2 were successfully transduced into DMD pericytes

Cells derived from the skeletal muscle of a DMD patient^6^ were transduced with lentivirus coding for mini-dystrophin constructs SFFV-C1-GFP, hDesmin-C1-GFP, SFFV-C2-GFP, and hDesmin-C2-GFP using an MOI of 10 (according to the proviral copy number). Lentiviruses coding for hDesmin-GFP or SFFV-GFP were used as controls. The transduction efficiency was examined by FACS, 7 days after transduction (Figure S1). There were 59.3%, 6.53%, 71.3% and 50.2% GFP+ cells within SFFV-C1-GFP, hDesmin-C1-GFP, SFFV-C2-GFP and hDesmin-C2-GFP transduced cells, respectively, and 92.6% and 87.9% GFP+ cells within SFFV-GFP and hDesmin-GFP lentivirus-transduced cells.

### Dystrophin protein is produced in transduced non-differentiated cells

**Figure 2:**
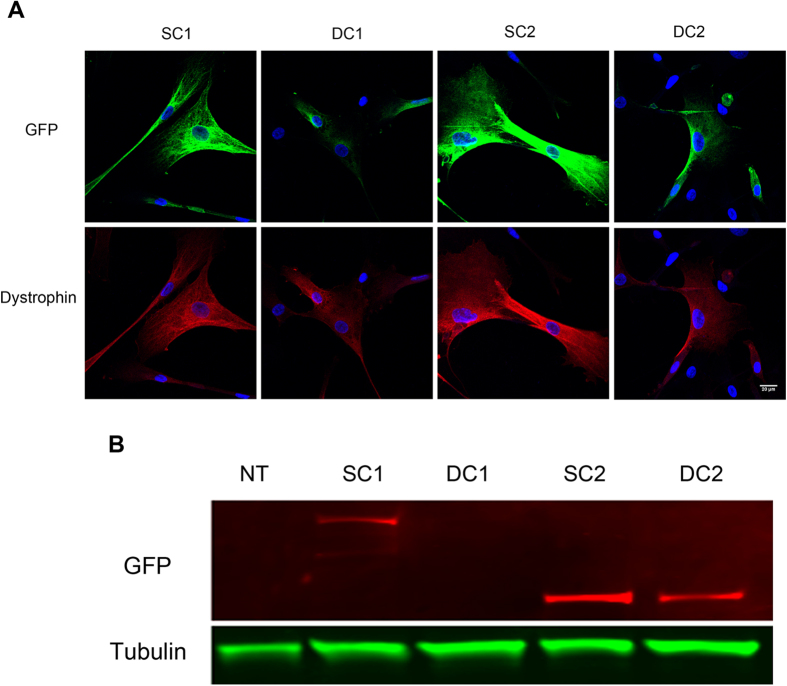
Expression of dystrophin in lentivirally-transduced pericytes. (**A**) Confocal images of DMD pericytes that had been transduced with dystrophin lentivirus. GFP+ cells were FACS sorted and plated in culture, then fixed with 4% paraformaldehyde and stained with GFP (green) and dystrophin (red). Nuclei were counterstained with DAPI (blue). Scale bar = 20 μm. SC1: SFFV-C1-GFP, DC1: hDesmin-C1-GFP, SC2: SFFV-C2-GFP and DC2: hDesmin-C2-GFP. (**B**) Western blotting of GFP (upper lane) and Tubulin 2.1 (lower lane) on samples extracted from DMD pericytes which had been transduced with SFFV-C1-GFP (SC1); hDesmin-C1-GFP (DC1); SFFV-C2-GFP (SC2) and hDesmin-C2-GFP (DC2) lentivirus. GFP+ cells were purified by FACS before sample preparation. Sample extracted from non-transduced (NT) pericytes was loaded as negative control. This is a cropped image; the full-length blot is presented in Supplementary Figure S2.

GFP+ cells were purified by FACS and expanded *in vitro* for analysis. Double staining with GFP and dystrophin antibodies confirmed that they were, as expected, co-localized in cells transduced with SFFV-C1-GFP, hDesmin-C1-GFP, SFFV-C2-GFP and hDesmin-C2-GFP lentiviruses (Fig. 2A). We could therefore use GFP as surrogate for dystrophin expression. GFP was expressed at different intensities within SFFV-C1-GFP+, hDesmin-C1-GFP+, SFFV-C2-GFP+ and hDesmin-C2-GFP+ cell populations, suggesting that different virus copy numbers had integrated into each individual cell. An alternative explanation for the lower expression of dystrophin-GFP driven by the hDesmin promoter could be lower activity of the hDesmin promoter than the SFFV promoter in the context of the study. GFP was present within the cytoplasm in all groups of non-differentiated cells. Within CD133+ cells derived from a normal donor^7^, no dystrophin was present within non-differentiated cells (data not shown).

Dystrophin expression in transduced cells was verified by western blotting analysis using a GFP antibody (Fig. 2B) or dystrophin antibody (supplementary data Figure S3 and S4). The staining pattern of the two antibodies was identical, therefore we used GFP as surrogate of dystrophin expression. Expression of C1 was barely detectable in hDesmin-C1-GFP transduced cells, whilst there were clear bands in SFFV-C1-GFP, SFFV-C2-GFP and hDesmin-C2-GFP transduced cells.

### Dystrophin protein is expressed in cells undergoing myogenic differentiation *in vitro*

**Figure 3:**
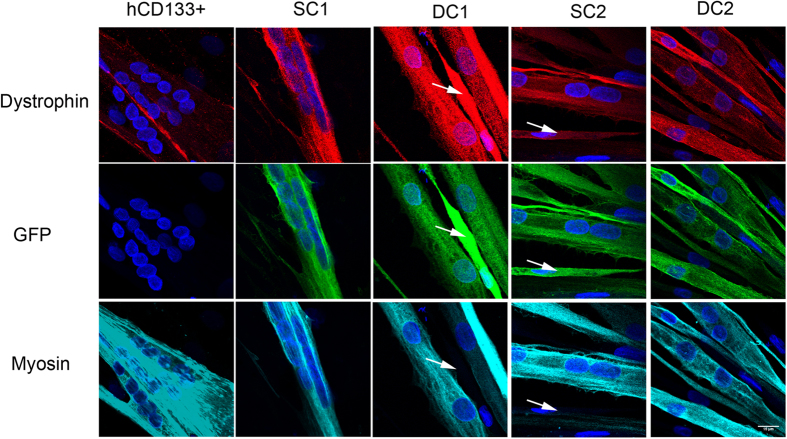
Expression of dystrophin in differentiating pericytes that had been transduced with SFFV-C1-GFP (SC1); hDesmin-C1-GFP (DC1); SFFV-C2-GFP (SC2) and hDesmin-C2-GFP (DC2) lentivirus. Purified GFP+ cells from each cell preparation were induced to differentiate in culture for 7 days, and stained with antibodies to GFP (green), dystrophin (red) and myosin (MF20, cyan). hCD133+ cells derived from a normal donor were treated in parallel as positive control for the dystrophin staining. White arrows point to cells that express GFP and dystrophin, but did not express myosin and were not differentiated multinucleated myotubes. Nuclei were counterstained with DAPI (blue). Scale bar = 15 μm.

Full-length dystrophin was located in differentiated myotubes derived from normal CD133+ cells; no dystrophin was present in non-differentiated cells within this culture (Fig. 3). However, within differentiated cultures derived from DMD pericytes that had been transduced with dystrophin C1 or C2 constructs, dystrophin-GFP was found within both myotubes and non-differentiated cells (Fig. 3).

Myotubes derived from normal CD133+ cells (thus expressing full length dystrophin driven by the endogenous dystrophin promoter) had diffuse low fluorescent intensity within their cytoplasm, with stronger punctate signal along the membrane. However, dystrophins C1 and C2 were expressed throughout the cytoplasm of the myotube, with slightly stronger and punctate signal pattern at the membrane (Fig. 3).

### SFFV-C1-GFP, SFFV-C2-GFP and hDesmin-C2-GFP were expressed within the majority of fibres of donor origin *in vivo*

As the transduction efficiency of lentiviruses SFFV-C1-GFP, SFFV-C2-GFP and hDesmin-C2-GFP was high (more than 50% GFP+ cells), while the transduction efficiency of lentivirus hDesmin-C1-GFP was far lower (only 6.53% GFP+ cells) (Figure S1), we therefore FACS sorted GFP+ cells from the hDesmin-C1-GFP transduced population, and transplanted them in parallel with non-sorted SFFV-C1-GFP, SFFV-C2-GFP and hDesmin-C2-GFP cells into recipient mice.

**Figure 4:**
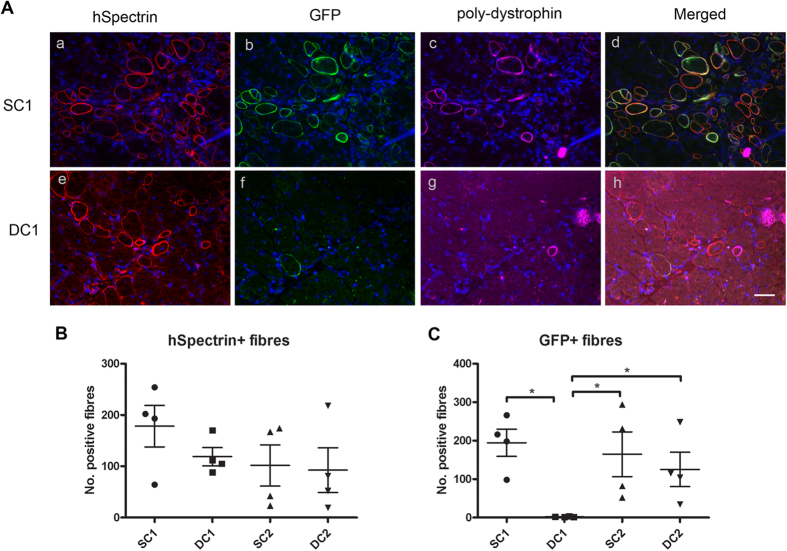
Restoration of dystrophin in regenerated muscle fibres derived from lentivirally-transduced DMD pericytes. (**A**) shows representative transverse sections of cryodamaged muscles of an mdx nude mouse that had been grafted with a–d: SFFV-C1-GFP cells; e–h: hDesmin-C1-GFP cells. Muscles transplanted with SFFV-C2-GFP and hDesmin-C2-GFP cells showed similar staining pattern as that shown in a–d. Sections were stained with antibodies to human spectrin (red), GFP (green), dystrophin (cyan). Nuclei were counterstained with DAPI (blue). Scale bar = 15 μm. (**B**) and (**C**) are the quantification of human Spectrin+ fibres (**B**) and GFP+ fibres (**C**) in SFFV-C1-GFP, hDesmin-C1-GFP, SFFV-C2-GFP and hDesmin-C2-GFP cells transplanted groups. *p < 0.05.

One month after intra-muscular transplantation, donor-derived muscle fibres (expressing human spectrin) were present in all of the transplanted muscles. Co-staining of GFP and dystrophin showed that in muscles transplanted with SFFV-C1-GFP, SFFV-C2-GFP and hDesmin-C2-GFP cells, the majority of the human spectrin+ fibres expressed dystrophin and GFP (Fig. 4A a–d); while in muscles transplanted with hDesmin-C1-GFP+ cells, there were similar numbers of fibres expressing human spectrin, but only a few of these expressed dystrophin (Fig. 4A e–h). The number of human spectrin+ fibres (mean ± SEM) was 178.3 ± 40.39, 118.8 ± 17.81, 101.5 ± 40.05 and 92.50 ± 43.71 in SFFV-C1-GFP, hDesmin-C1-GFP, SFFV-C2-GFP and hDesmin-C2-GFP cell transplanted groups, respectively (Fig. 4B), there were no differences among these groups (*p* = 0.3910, one way ANOVA). However, the number of GFP+ fibres (mean ± SEM) was 194.5 ± 35.24, 1.750 ± 0.4787, 164.8 ± 58.21 and 125.3 ± 44.68 in SFFV-C1-GFP, hDesmin-C1-GFP, SFFV-C2-GFP and hDesmin-C2-GFP cell transplanted groups (Fig. 4C); there were significant differences in the number of GFP+ fibres between SFFV-C1-GFP and hDesmin-C1-GFP (*p* = 0.0286, Mann-Whitney test), SFFV-C2-GFP and hDesmin-C1-GFP (*p* = 0.0294, Mann-Whitney test) as well as hDesmin-C2-GFP and hDesmin-C1-GFP (*p* = 0.0294, Mann-Whitney test) groups, suggesting that hDesmin-C1-GFP was not expressed *in vivo*.

In transplanted muscles, all GFP+ fibres were dystrophin+, but not all dystrophin+ fibres expressed GFP. This was because the dystrophin antibody that we used recognizes revertant muscle fibres that occur naturally in mdx muscles^20^,^21^,^22^. GFP is therefore a better marker for donor fibres than is dystrophin and we used GFP in subsequent experiments as a surrogate for donor-derived dystrophin.

### Components of the dystrophin-associated glycoprotein complexes (DGC) are recruited in muscle fibres expressing dystrophins C1 or C2.

To determine whether the dystrophin expressed within regenerated muscle fibres *in vivo* was functional, we investigated whether the dystrophin-associated glycoprotein complex (DGC) was restored within these muscle fibres^23^. Both C1 and C2 contain the cysteine-rich domain, which are the binding sites for β-dystroglycan^24^,^25^, a major component of the DGC.

**Figure 5:**
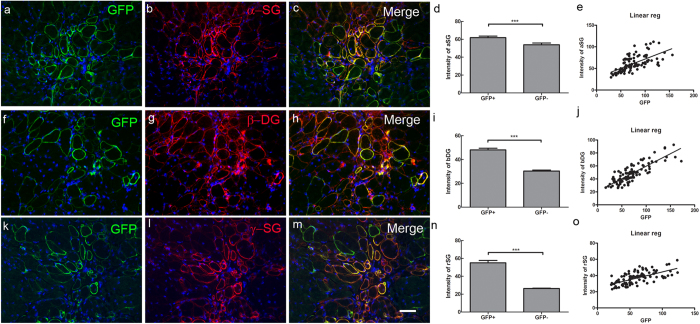
Recruitment of members of the DGC in dystrophin-expressing fibres of donor origin. Representative transverse sections of muscles that had been transplanted with SFFV-C1-GFP donor cells, stained with antibodies to: GFP (green, **a**, **f, k**), α-sarcoglycan (α-SG, red, **b**), β-dystroglycan (β-DG, red, **g**) and γ-sarcoglycan (γ-SG, red, l). **c, h** and **m** are merged images of **a** and **b; f** and **g; k** and **l**, respectively. Nuclei were counterstained with DAPI (blue). Scale bar = 15 μm. Muscles that had been transplanted with SFFV-C2-GFP, hDesmin-C2-GFP cells (not shown here) showed a similar staining pattern to SFFV-C1-GFP cell transplanted muscles. **d, i** and **n**: expression intensity of α-sarcoglycan (**d**), β-dystroglycan (**i**) and γ-sarcoglycan (**n**) was significantly higher in GFP+ compared to GFP- fibres in representative muscles that had been transplanted with SFFV-C1-GFP, SFFV-C2-GFP and hDesmin-C2-GFP cells. The intensity of expression of each component of the DGC correlated positively to GFP intensity (**e, j** and **o**). ***p < 0.0001.

There was weak expression of the DGC components α-sarcoglycan, β-dystroglycan and γ-sarcoglycan on the sarcolemma of myofibres in non-transplanted mdx nude mouse muscles (data not shown), similar to mdx mice^26^,^27^,^28^. However, similar to regenerated muscles derived from normal CD133+ cells (data not shown), muscles that had been grafted with SFFV-C1-GFP (Fig. 5) SFFV-C2-GFP, and hDesmin-C2-GFP cells (data not shown) showed up-regulation of α-sarcoglycan, β-dystroglycan and γ-sarcoglycan in dystrophin+ fibres. There was a positive correlation between the expression intensity of these DGC proteins and the intensity of the GFP on the muscle fibres. There were almost no fibres expressing donor-derived dystrophin-GFP in muscles that were grafted with hDesmin-C1-GFP+ cells (Fig. 4), and no DGC restoration in these muscles.

### Recruitment of neuronal nitric oxide synthase (nNOS) on SFFV-C1-dystrophin expressing fibres

**Figure 6:**
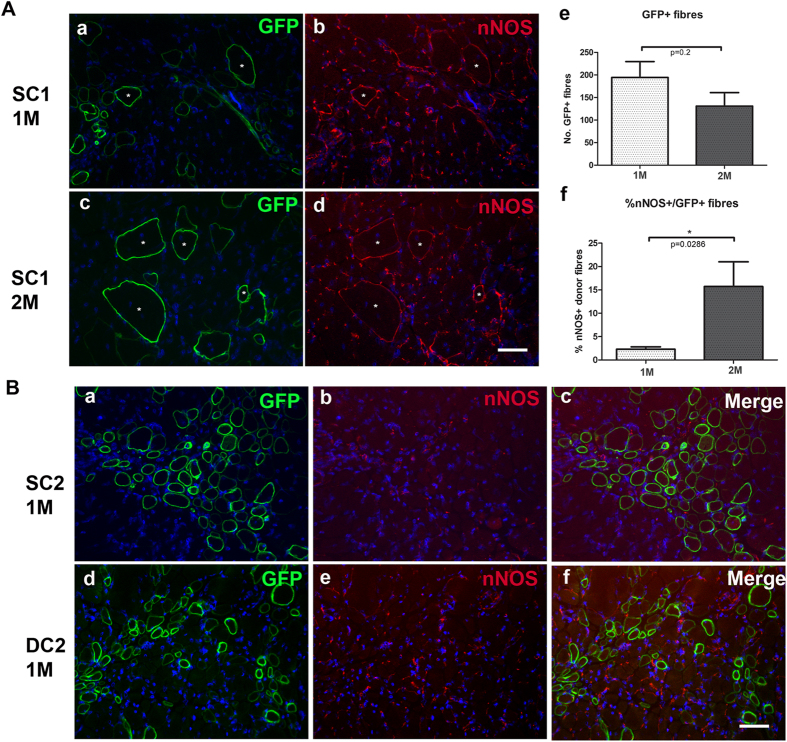
Recruitment of nNOS in dystrophin- expressing regenerated myofibres *in vivo*. (**A**) Recruitment of nNOS in SFFV-C1-GFP- expressing regenerated myofibres *in vivo*. (a–d): Fibres expressing nNOS (red, b, d) that were also GFP+ (green, a, c) on a representative transverse section of host muscle that had been cryodamaged and transplanted with SFFV-C1-GFP cells one month (a,b) or two months (c,d) previously. Nuclei were counterstained with DAPI (blue). Asterisks (*) indicate nNOS+/GFP+ fibres. Scale bar = 15 μm. (e) quantification of the number of GFP+ fibres in muscles that had been transplanted with SFFV-C1-GFP cells one or two months previously. There was no significant difference in the number of GFP+ fibres between the two time points, *p* = 0.2. (f) percentage of the GFP+ fibres that expressed nNOS. There was a significantly higher percentage of dystrophin- expressing fibres of donor origin that also expressed n-NOS, at two months than at one month after transplantation. *p = 0.0286. (**B**) nNOS was not expressed on regenerated muscle fibres of donor origin expressing either SFFV-C2-GFP (SC2) or hDesmin-C2-GFP (DC2). (a–c) double staining with antibodies to GFP (a) and nNOS (b) on a representative transverse section of muscle that had been transplanted with SC2 cells. (c) merged image of a and b. Nuclei were counterstained with DAPI (blue). Scale bar = 15 μm. (d–f) double staining with antibodies to GFP (d) and nNOS (e) on a representative transverse section of muscle that had been transplanted with DC2 cells. (f) merged image of d and e. Nuclei were counterstained with DAPI (blue). Scale bar = 15 μm.

nNOS was absent at the sarcolemma of muscle fibres (apart from revertant fibres) of mdx nude mice, but was expressed on blood vessels (data not shown)^29^,^30^. To verify whether the presence of the nNOS binding motif (R16/17 of the spectrin repeats) on dystrophin C1 could effectively recruit nNOS to muscle fibres, we performed immunostaining of nNOS and GFP on muscle sections that had been grafted with transduced cells. The expression of nNOS was examined at one and two months after transplantation. To exclude the “host derived” nNOS on revertant, dystrophin+ fibres^30^, we only counted nNOS+/GFP+ fibres within the grafted muscles. There were no nNOS+/GFP+ fibres in muscles that had been transplanted with hDesmin-C1-GFP, SFFV-C2-GFP and hDesmin-C2-GFP cells (Fig. 6B). In all muscles that had been transplanted with SFFV-C1-GFP cells, nNOS+/GFP+ fibres were found. One month after cell transplantation, within 194.5 ± 35.24 GFP+ donor-derived fibres, there were 4.75 ± 1.80 nNOS+/GFP+ fibres comprising 2.303 ± 0.51% of the total GFP+ fibres. Two months post-transplantation, within 131.0 ± 30.0 GFP+ donor-derived fibres, there were 16.75 ± 3.40 nNOS+/GFP+ fibres (15.72 ± 5.29% of the total GFP+ fibres) (mean ± SEM, n = 4 muscles for each time point). There was no significant difference in the number of total GFP+ donor-derived fibres between one and two month after transplantation (Fig. 6A–e); however, there was a significantly higher percentage of dystrophin expressing fibres of donor origin expressing nNOS (nNOS+ /GFP+ fibres) at two than at one month after cell transplantation (*p* = 0.0256, Fig. 6A–f).

## Discussion

In our cell transplantation mouse model, we have restored functional dystrophin in regenerated muscle fibres derived from DMD patient-derived stem cells that had been lentivirally-transduced with two novel dystrophin constructs. We further show that dystrophin transgene C1 which is larger than those that have been tested previously, can be put into a lentiviral vector, used to transduce human stem cells that, following their transplantation into dystrophin-deficient mouse muscle, give rise to functional dystrophin protein.

Although insertional mutagenesis does not seem to cause problems in some preclinical models for hematopoietic stem cells^31^,^32^, it does remain a concern for clinical application of lentiviruses. However, there are other ways of reducing the risk of insertional mutagenesis^33^,^34^.

When generating the dystrophin lentiviruses, one problem we encountered was the effect of the insert size on the virus titre. It has been shown that lentiviruses are limited by the length of RNA that they can package, which is generally thought to drop at an increasing rate above a provirus length of 11 kb^10^,^17^. It is noteworthy that some of the dystrophin lentiviruses used in this investigation were in excess of this limit. The dystrophin constructs, when fused to EGFP and driven by the 1.8 kb hDesmin promoter, give a genomic RNA length of 12.851 kb for C1 (ΔR3R13) and 9.727 kb for C2 (ΔH2R23). These genomic RNA lengths are either in excess of, or just at the upper limit of lentiviral packaging capacity, with the length of the hDesmin-C1-GFP construct appearing to be incompatible with this gene transfer system. In order to enable efficient packaging of C1 and C2 into lentiviruses, these dystrophin constructs were also packaged into vectors driven by the SFFV promoter, which is approximately 1.4 kb smaller than the hDesmin promoter. The lentiviral genomic RNA of these constructs is 11.266 kb for SFFV-C1-GFP and 8.134 kb for SFFV-C2-GFP, which facilitated their application to this technology.

The effect of the insertion size on the lentivirus packaging efficiency was evident in both the transduction and transplantation experiments. As to the largest dystrophin construct, hDesmin-C1-GFP, we were unable to produce a virus of sufficient titre to transduce mdcs under the same conditions as the other treatments (supplementary Figure S1). Prior to transplantation into mdx mice, these cells were purified on the basis of GFP expression, to maximise the delivery of dystrophin-expressing cells. However, one month after transplantation, the majority of the donor muscle fibres were not expressing dystrophin or GFP (Fig. 4), which suggested that the expression of hDesmin-C1-GFP was not retained in the cells.

However, the delivery of dystrophin C1 was markedly improved when driven by the smaller sized SFFV promoter (supplementary Figure S1). The level of the transduction efficiency was equivalent to that of hDesmin-C2-GFP lentivirus and the integration of SFFV-C1-GFP, SFFV-C2-GFP and hDesmin-C2-GFP lentivirus appeared to be stable, given that nearly all of the donor fibres expressed dystrophin and GFP one month after transplantation (Fig. 4).

When the GFP+ cells were purified by FACS sorting and examined for their expression of dystrophin by immunostaining, all of the transduced groups contained cells expressing dystrophin. This is not unexpected, as transgene expression was under the control of either SFFV or hDesmin promoter; the SFFV promoter is ubiquitous and the hDesmin promoter is expressed in myogenic cells that have not yet begun terminal differentiation^16^, as well as in differentiated myotubes and muscle fibres. This explains the strong expression of these dystrophins in the cytoplasm of both non-differentiated and differentiated cells. Nevertheless, these cells formed muscle after their transplantation *in vivo*, evidence that their muscle regenerative capacity was not noticeably compromised. In these regenerated muscle fibres, the dystrophin derived from the donor myonuclei was correctly localised at the sarcolemma of the muscle fibres. It should be noted that our cells contained a dystrophin-GFP fusion protein and although GFP itself may have a deleterious effect on the function of some types of cell^35^,^36^, it did not appear to affect the function of the skeletal muscle derived stem cells used in our study. A recent publication^37^ has indicated that dystrophin is expressed in satellite cells and is important for regulating their asymmetric division, so expression of dystrophin within non-differentiated muscle stem cells derived from DMD patients might improve, rather than be deleterious to, their function.

Systemic delivery of autologous genetically-modified stem cells would be ideal for the treatment of DMD. However, using the same cells and mouse model that we use in this study, we found that cells delivered intra-arterially did not give rise to muscle in downstream muscles, but when delivered intra-muscularly, they contributed robustly to muscle regeneration^6^. This is in contrast to the findings of Dellavalle *et al*.^5^, using similar human cells and mdx host mice on a different immunodeficient background (discussed in Meng 2011^6^). So, in this proof of principle study, we chose to use a delivery route (intramuscular delivery) that is effective for our cells and mouse model, in order to investigate the effects of the two novel dystrophin constructs.

In these experiments, the levels of donor cell engraftment are not high enough to effect physiological improvement. Although some publications have reported increase in muscle strength in dystrophin-deficient mouse muscles following very low-level engraftment of donor cells^38^, more recent work has indicated that there must be a minimum level of restored dystrophin to confer functional benefit to the muscle^39^,^40^. Using a mouse model with varying, low dystrophin levels due to skewed X-inactivation, it was shown that 20% of normal levels of dystrophin are needed to fully protect muscle fibers from exercise-induced damage^41^ . A recent paper shows that 15% of normal levels of dystrophin, homogenously located at the sarcolemma of muscle fibres throughout the muscle, are required in an mdx tibialis anterior muscle to protect against eccentric contraction-induced damage^42^. There was also a positive correlation between maximal specific force and dystrophin expression in muscles in which restored dystrophin was homogeneously expressed^42^. However, if not all fibres within a treated muscle are expressing dystrophin, the picture is rather different: indeed 65% of muscle fibres expressing an internally-deleted dystrophin protein provided protection against eccentric contraction-induced damage in the TA of mdx muscle^43^. Mouse satellite cells that had been transplanted intra-muscularly in a new, highly immunodeficient mdx mouse model gave rise to more than 1,000 dystrophin+ fibres in the transplanted muscles. These muscles had significantly greater maximal tetanic force and specific force than PBS-injected control muscles^44^. But in our experiments described here in which we had transplanted human pericytes into mdx nude host mice, dystrophin was only present in fewer than 200 fibres within transplanted muscles. We therefore decided not perform physiological measurements of our transplanted muscles, as the dystrophin was clearly below levels required to have a meaningful clinical benefit. Instead, we investigated whether members of the DGC and nNOS were restored in muscle fibres expressing restored dystrophin, which is evidence that the dystrophin was functional^45^.

The transplantation efficiency of the SFFV-C1-GFP, SFFV-C2-GFP and hDesmin-C2-GFP cells was similar. Dystrophin restoration and recruitment of DGC within myofibres of donor origin was also comparable in grafts transplanted with cells expressing all three constructs (Figs 4 and 5). However, an important difference between regenerated myofibres expressing either C1 or C2 dystrophin was in the recruitment of nNOS. As expected, there was nNOS on donor fibres derived from SFFV-C1-GFP cells, but not on regenerated myofibres derived from either SFFV-C2-GFP or hDesmin-C2-GFP expressing cells (Fig. 6). This is not surprising, as the C1 construct contains the rod domain of the nNOS binding site, which enables recruitment of nNOS on the dystrophin -expressing fibres^13^. Dystrophin C1 is therefore a novel dystrophin construct that can successfully recruit nNOS expression on muscle fibres *in vivo*. Furthermore, the number of nNOS+/GFP+ fibres significantly increased with time post transplantation, in line with previous observations suggesting that the sarcolemmal localisation of nNOS may depend on the maturation of the muscle fibres^46^.

Our study provides proof of principle that a dystrophin gene introduced by a lentivirus into human skeletal muscle derived cells can mediate expression of functional dystrophin. The transplantation of autologous cells, engineered to express dystrophin, would be a potential therapeutic tool to treat a broad spectrum of DMD patients with different mutations. We report the application of two novel functional mini-dystrophin constructs in this system. In addition, our findings support the advantage of using the larger, more functional dystrophin C1, which contains the nNOS binding site, in combination with DMD muscle cells. The formation of functional dystrophin C1 protein, which recruits nNOS on muscle fibres in the mdx nude muscle environment, provides evidence that this novel construct C1 is a promising dystrophin construct with potential to be used in autologous stem cell transplantation to treat DMD.

It might be possible to combine AAV-mediated gene therapy with lentivirally-mediated stem cells for dystrophin restoration. AAV coding for microdystrophin can be delivered systemically^47^,^48^. AAV targets postmitotic muscle fibres, but not quiescent satellite cells^49^. Satellite cells are needed to maintain the myofibres; systemically delivering AAV to the patients would leave the satellite cells untreated, which would lead to a net loss of dystrophin in muscle fibres with time. If AAV-dystrophin treated patients were also treated with autologous stem cells coding for dystrophin, the stem cells should be able to repair and maintain muscle fibres and lead to continued dystrophin expression throughout life. However, consideration would have to be given to the promoter and dystrophin constructs used for this strategy, particularly as lentivirus accommodates our C1 and C2 constructs, whereas AAV would not.

## Materials and Methods

### Ethics

Human cells were obtained from the MRC Centre for Neuromuscular Diseases Biobank. Tissue sampling was approved by the NHS National Research Ethics Service, Hammersmith and Queen Charlotte’s and Chelsea Research Ethics Committee: Setting up of a Rare Diseases biological samples bank (Biobank) for research to facilitate pharmacological, gene and cell therapy trials in neuromuscular disorders (REC reference number 06/Q0406/33) and the use of cells as a model system to study pathogenesis and therapeutic strategies for Neuromuscular Disorders (REC reference 13/LO/1826), in compliance with national guidelines regarding the use of biopsy tissue for research. All patients or their legal guardians gave written informed consent.

Mdx nude mice were bred and experimental procedures were carried out in the Biological Services Unit, University College London Institute of Child Health, in accordance with the Animals (Scientific Procedures) Act 1986. Experiments were approved by the UCL Animal Welfare and Ethical Review Body. Experiments were performed under Home Office licence number 70/7086.

### Isolation and maintenance of human skeletal muscle derived pericytes

Human skeletal muscle derived cells (pD2) isolated from the extensor digitorum brevis muscle of an 11 year old DMD patient were used. These cells contribute to robust regeneration after intra-muscular transplantation into muscles of immunodeficient host mice^6^. Cells were maintained in M10 medium (Megacell DMEM (Sigma, Dorset, UK) medium, 10% fetal bovine serum (Invitrogen, Paisley, UK), 2 μM glutamine (Sigma, Dorset, UK), 1% non-essential amino acids (Sigma, Dorset, UK), 0.1 mM β- mercaptoethanol (Sigma, Dorset, UK) and 5 ng/ml basic fibroblast growth factor (PeproTech, London, UK). For transduction of dystrophin lentivirus, cells at a mean population doubling of 5.64 or 13.13 were used.

### Vector construct and preparation

The 7.4 kb C1/ΔR3-R13 and the 4.2 kb C2/ΔH2-R23 mini-dystrophins (see detailed sequence in Supplementary data) were generated by splicing by overlapping extension (SOE-PCR) using human full-length dystrophin cDNA as template. The pRRL.sin.cppt.desmin.GFP construct^15^,^16^ was kindly provided by Michael Antoniou. The construction of pRRL.desmin.C2 was carried out by GeneArt cloning services (Life Technologies, Paisley, UK) (supplementary methods), and pRRL.desmin.C1 was constructed by cloning the respective regions from pCMV. 3ΔR3R13 into the pRRL.desmin.C2 construct. The GFP tag was fused to each vector using a standard cloning strategy. For promoter comparison, a SFFV-promoter was also cloned into the lentiviral vector backbone to generate SFFV-C1-GFP and SFFV-C2-GFP vectors.

Third-generation, VSV-G-pseudotyped lentiviral vectors were produced by co-transfecting the respective dystrophin plasmid into HEK 293T cells along with pMD2G, pRRE.MDL2 and pRSV.REV (a kind gift from Kathryn North)^50^,^51^. Plasmids were transfected in a 4:2:1:1 ratio, based on the molar mass of each plasmid. Vectors were purified by low-speed ultracentrifugation at 20,000xg over a sucrose cushion, as described previously^10^.

### Viral titration

Vectors were titred by viral gRNA copy number, proviral copy number, integrated proviral copy number as well as functional titre (FACS) levels (supplementary methods).

### FACS sorting and analysis

7 days after transduction, cells were trypsinized and 5 × 10^4^ cells were used for analysis of GFP expression using a LSRII FACS machine (BD Biosciences, Oxford, UK). Non-transduced cells were used to set the negative control gate. For *in vitro* analysis, GFP+ cells from cells transduced with each promoter/dystrophin construct (Figure S1) were sorted using a Moflo FACS sorting machine, and expanded *in vitro* for further analysis of dystrophin expression. All the FACS data were analyzed using Flowjo 7.2.5 software.

### Immunofluorescence staining of dystrophin/GFP on cells and myotubes

After FACS sorting, GFP+ cells were expanded *in vitro* and were plated onto 50 μg/ml poly-D-lysine coated 8-well chamber slides (Fisher Scientific, Loughborough, UK) in M10 medium at a density of 2 × 10^4^ cells/well. Cells were fixed 24 hours after plating with 4% paraformaldehyde (PFA) (Sigma, Dorset, UK) for 15 min at room temperature.

To investigate the expression of dystrophin in differentiated myotubes, GFP+ cells from each preparation were plated onto 0.1 mg/ml Matrigel (BD Bioscience, Oxford, UK)-coated 8 well chamber slides in M10 medium at a density of 5 × 10^4^ cells /well. Medium was changed into skeletal muscle cell differentiation medium (Promocell, Heidelberg, Germany) 24 hours after plating to induce myogenic differentiation. Cells were fixed with 4% PFA 7 days later. Pericytes derived from normal human muscle would have been an ideal positive control, but for the fact that our preparations from normal muscle are not very myogenic (see Meng, 2011, Table 1)^6^. We therefore chose a preparation of normal human skeletal muscle-derived CD133+ cells^7^ that are as highly myogenic as the pericytes derived from the skeletal muscle of a DMD patient that we used in this study, as the positive control to show dystrophin in myotubes *in vitro*.

Fixed cells were blocked with 10% normal goat serum and 0.03% Triton X100 in PBS (blocking solution) for 1 hour at room temperature. Chicken anti-GFP (1:1000, Abcam, Cambridge, UK), rabbit anti-dystrophin (1:1000, Fisher Scientific, Loughborough, UK) and mouse anti-myosin (MF20, 1:100, DSHB, Iowa City, Iowa) antibodies diluted in blocking solution were then applied for 2 hours at room temperature. Alexa-488 conjugated goat anti-chicken IgG (H + L) antibody (1:500, Invitrogen, Paisley, UK), 594-conjugated goat anti-rabbit IgG (H + L) antibody (1:500, Invitrogen, Paisley, UK) and 647-conjugated goat anti-mouse IgG2b antibody (1:500, Invitrogen, Paisley, UK) were then applied for 1 hour at room temperature before adding mounting medium (DAKO, Ely, UK) containing 10 μg/ml 4′,6-diamidino-2-phenylindole (DAPI). Images were captured with a Zeiss confocal microscope.

### Western blotting

To visualize the expression of dystrophin after transduction, GFP+ cells from each transduced population were plated onto 1 mg/ml Matrigel-coated 10 cm^2^ culture dishes (Fisher Scientific, Loughborough, UK) at a density of 1 × 10^6^ cells /dish in M10 medium. Samples were collected 24 hours after culture. Protein was extracted in RIPA (Radio-Immunoprecipitation Assay) lysis buffer (Sigma, Dorset, UK) containing a complete protease inhibitor cocktail (1:100, Roche, Welwyn Garden City, UK). 100 μl of lysis buffer was added to each 10 cm^2^ culture dish, and left on ice for 10 minutes. Samples were collected and boiled for 3 minutes and then centrifuged at 14,000 g for 10 minutes at 4 °C. The supernatants were stored at −20 °C until needed. 30 μl /well of each sample were loaded onto NuPAGE Novex 3–8% Tris-Acetate Gel, and run at a constant voltage of 150 V for 1 hour, before being transferred to a nitrocellulose membrane at 300 mA for 2 hours. The membrane was then blocked with Odyssey block solution (LI-COR Biosciences, Cambridge, UK) for 60min, incubated by a cocktail of primary antibodies of rabbit anti-GFP antibody (1:2000, Invitrogen, Paisley, UK) (or rabbit anti-dystrophin antibody, 1:2000, Fisher Scientific, Loughborough, UK) and mouse anti-tubulin (1:2000, Invitrogen, Paisley, UK) overnight at 4 °C. After washing with PBS containing 1% Tween 20 (PBST) for 15 min × 3 times at room temperature, the membrane was then incubated with IRDye 680 RD goat anti-rabbit and IRDye 800CW goat anti-mouse 2^nd^ antibodies (1:15000, LI-COR Biosciences, Cambridge, UK) for 1–2 hrs at RT. The image of the blotted membrane was acquired by Odyssey Clx infrared imaging system (LI-COR Biosciences, Cambridge, UK) using image studio software 3.1.4.

### *In vivo* transplantation and analysis

#### Intra-muscular transplantation of dystrophin transduced human pericytes.

4–8 week old mdx nude mice were used as recipients in this study. On the day of transplantation, mice were anaesthetized with isofluorane and tibialis anterior (TA) muscles of both hindlimbs were injured by cryoinjury as described previously^52^,^53^. 5 × 10^5^ cells in 5 μl culture medium were then injected into each TA with a Hamilton syringe. The skin was sutured after injection and the mice were kept warm until they had fully recovered from the anaesthetic. For analgesia, Vetergesic (buprenorphine hydrochloride; 0.05 mg/kg body weight; Reckitt and Coleman, London, UK) was injected subcutaneously.

TA muscles were transplanted with pericytes that had been transduced with SFFV- C1-GFP, SFFV-C2-GFP, hDesmin C1-GFP and hDesmin-C2-GFP. In the hDesmin-C1 group, GFP+ cells purified by FACS sorting were used for grafting. Muscles were removed for analysis one month (n = 4 per group) after grafting. For examining the restoration of nNOS by donor fibres, muscles were analyzed at both one month (n = 4) and two months (n = 4) after grafting.

#### Analysis of muscle sections

Grafted TA muscles were dissected and frozen in isopentane pre-chilled in liquid nitrogen. 10 μm transverse cryosections were cut throughout the muscle and stained with antibodies to human spectrin (Vector labs, VP-S283, 1:100, Peterborough, UK), GFP (1:1000, chicken polyclonal, Abcam, Cambridge, UK), poly-dystrophin (1:1000, Fisher Scientific, Loughborough, UK) followed by corresponding secondary antibodies (Alexa 488 conjugated goat anti-chicken IgG (H + L), Alexa 594 conjugated goat anti-mouse IgG (H + L), Alexa 647 conjugated goat anti-rabbit IgG (H + L), Invitrogen, Paisley, UK). To investigate the expression of DGC and nNOS, serial sections were stained with antibodies against α-Sarcoglycan (Leica Biosystems, clone Ad1/20A6, 1:100, Newcastle upon Tyne, UK)/GFP, γ-Sarcoglycan (Source Bioscience, 1:500, Nottingham, UK)/GFP, β-dystroglycan (Leica Biosystems, clone 43DAG1/8D5, 1:100, Newcastle upon Tyne, UK)/GFP and nNOS (Santa Cruz, NOS1 (R-20), 1:500, Middlesex, UK)/GFP, followed by corresponding secondary antibodies as described above. The intensity of the DGC proteins on GFP+ fibres and GFP- fibres were measured using MetaMorph software and compared within each muscle. Images were captured with MetaMorph software using a Leica microscope. The number of GFP+ fibres and nNOS+ fibres were counted in sections that contained the most positive fibres, using MetaMorph software. The data were analyzed by Mann-Whitney test using graphpad prism5 software.

## Additional Information

**How to cite this article**: Meng, J. *et al*. Autologous skeletal muscle derived cells expressing a novel functional dystrophin provide a potential therapy for Duchenne Muscular Dystrophy. *Sci. Rep.* **6**, 19750; doi: 10.1038/srep19750 (2016).

## Supplementary Material

Supplementary Information
