# Supplementary material for: Autologous skeletal muscle derived cells expressing a novel functional dystrophin provide a potential therapy for Duchenne Muscular Dystrophy
Source: Sci Rep. 2016 Jan 27;6:19750. doi: 10.1038/srep19750 (PMC4728433; doi:10.1038/srep19750)

# Autologous skeletal muscle derived cells expressing a novel functional dystrophin provide a potential therapy for Duchenne Muscular Dystrophy

Jinhong Meng[1], John R Counsell[1,2,4], Mojgan Reza[3], Steven H Laval[3], Olivier Danos[2§], Adrian Thrasher[4], Hanns Lochmüller[3], Francesco Muntoni[1] and Jennifer E Morgan[1*]

## Supplementary material

### Cloning of mini-dystrophin constructs

The truncation in C1 comprises 3672 bases between nucleotide 1619 and 5290, and 6801 bases in C2 between nucleotide 2199 and 8999 (GenBank NM 004006) (Figure 1A, supplementary materials). The 7.4 kb C1/ΔR3-R13 and the 4.2 kb C2/ΔH2-R23 mini-dystrophins were generated by splicing by overlapping extension (SOE-PCR) using human full-length dystrophin cDNA as template. There are 2 polymorphisms in C1 constructs: G/A in position 142 of dystrophin Exon 59, which is a non-pathogenic SNP (rs 1800280), and an extra –GCC inframe insertion at position 31 of Exon 47, which predicted by Mutationtaster website to be polymorphism. (http://www.mutationtaster.org/cgi-bin/MutationTaster/MutationTaster69.cgi). There are also 2 polymorphisms in the C2 construct: G/A in position 142 of dystrophin Exon 59; C/T in position 160 of Exon 59 which is a variant with low frequency in the ExAC database, predicted to be polymorphism.

The amplification products and the vector were digested with restriction enzymes *NgoM*IV and *Cla*I, purified and ligated back into the cDNA. The resulting constructs were fully sequenced (MWG Biotech, Ebersberg, Germany) for verification of the correct sequence and reading frame.

**Generation of lentiviral vectors**

An *Age*I site was inserted into pCMV. Δ H2R23, immediately upstream of the dystrophin 5'UTR. pRRL.desmin.C2, was then assembled by cloning the C2 mini-dystrophin from the *Age*I-modified pCMV. Δ H2R23 into pRRL.sin.cppt.desmin.GFP using sites *Age*I-*Sal*I. pRRL.desmin.C1 was constructed by cloning the respective regions from pCMV. Δ R3R13 into the pRRL.desmin.C2 construct using *Nsi*I and *Sal*I restriction sites. A GFP tag was then introduced to all constructs by replacing the C-terminal region of each construct with that of pCihDystrophinGFP using the *BstB*I and *Sal*I restriction sites for C2 and the *BstE*II and *Sal*I sites for C1. For promoter comparisons, the SFFV promoter was PCR cloned from pRRL.sin.cPPT.SFFV.NIGW and inserted in place of the desmin promoter. The final clones were verified by DNA sequencing.

**Titration of lentiviruses**

Viral RNA titres were measured in the purified viral harvests by quantitative reverse transcription polymerase chain reaction (qRT-PCR) using a commercial kit (CloneTech cat no. 631235). Viral RNA was extracted directly from the viral preparation and DNase treated prior to qRT-PCR, which was then carried out in a single reaction. Complementary DNA was generated during an initial reverse transcription step (42°C 5 min, 95°C 10 sec), which was directly followed by real time PCR amplification (40 cycles of: 95°C 5sec; 60°C 30sec). The titre was then calculated by extrapolation of a standard curve generated from RNA standards

of known copy number.

Proviral titre was determined using genomic DNA extracted from transduced HeLa cells, adapted from a reported method [43]. Briefly, 1 x $10^5$ HeLa cells were plated into each well of a 6 well plate and transduced with a range of volumes of the concentrated lentivirus. After 72 hours, the transduced HeLa genomic DNA was extracted and analysed by SYBR Green amplified qPCR. The lentiviral copy number was determined by PCR directed against the LTR using the primers 5'-AGCTTGCCTTGAGTGCTTCAA-3' and 5'-AGGGTCTGAGGGATCTCTAGTTACC-3'. The proviral copy number was normalised to the total allelic copy number by PCR of human albumin using primers 5'-GCTGTCATCTCTTGTGGGCTGT-3' and 5'-ACTCATGGGAGCTGCTGGTTC-3'.

Integrated proviral copy numbers were calculated by Alu-PCR, adapted from a reported protocol [44]. Briefly, 10ng of virally-transduced HeLa gDNA was amplified using conditions (95°C 15 minutes; 25 cycles of 95°C 30 sec, 55°C 30 sec, 72°C 3min30sec; 72°C 10mins) and primers Alu1 (5'-TCC CAG CTA CTG GGG AGG CTG AGG-3'), Alu2 (5'-GCC TCC CAA AGT GCT GGG ATT ACA G-3') and M667-L (5'-ATG CCA CGT AAG CGA AAC TCT GGC TAA CTA GGG AAC CCA CTG-3) to amplify integrated lentiviruses. A control reaction was processed omitting the Alu1 and Alu2 primers to account for any linear, single-stranded amplification from the M667-L oligo. The resulting amplicons were then diluted 1/100 in water and 5μl was used in a nested qPCR reaction which was amplified with a SYBR Green master mix under conditions (95°C 15 minutes; 25 cycles of 95°C 30 sec, 55°C 30 sec, 72°C 3min30sec; 72°C 10mins) and the primers AA55M (5'-GCT AGA GAT TTT CCA CAC TGA CTA A-3') and Lamda T (5'-ATG CCA CGT AAG CGA AAC T-3') to detect the incidence of LTR copies in the products. The relative level of integrated copies were calculated by $2^{-\Delta\Delta ct}$, in which the ct value for Alu-containing reactions was subtracted

from the control reaction, before this value was subtracted from the non-transduced sample value. The integrated proviral titre was then expressed relative to a sample of known titre.

The functional titre of the lentivirus was determined according to GFP output by transducing HeLa cells with serial dilutions of vector preparations and measuring the percentage of GFP positive cells by flow cytometry.

**Sequence of C1 construct (from ATG to EGFP)**

ATGCTTTGGTGGGAAGAAGTAGAGGACTGTTATGAAAGAGAAGATGTTCAAAAG
AAAACATTCACAAAATGGGTAAATGCACAATTTTCTAAGTTTGGGAAGCAGCAT
ATTGAGAACCTCTTCAGTGACCTACAGGATGGGAGGCGCCTCCTAGACCTCCTCG
AAGGCCTGACAGGGCAAAAACTGCCAAAAGAAAAAGGATCCACAAGAGTTCAT
GCCCTGAACAATGTCAACAAGGCACTGCGGGTTTTGCAGAACAATAATGTTGATT
TAGTGAATATTGGAAGTACTGACATCGTAGATGGAAATCATAAACTGACTCTTGG
TTTGATTTGGAATATAATCCTCCACTGGCAGGTCAAAAATGTAATGAAAAATATC
ATGGCTGGATTGCAACAAACCAACAGTGAAAAGATTCTCCTGAGCTGGGTCCGA
CAATCAACTCGTAATTATCCACAGGTTAATGTAATCAACTTCACCACCAGCTGGT
CTGATGGCCTGGCTTTGAATGCTCTCATCCATAGTCATAGGCCAGACCTATTTGA
CTGGAATAGTGTGGTTTGCCAGCAGTCAGCCACACAACGACTGGAACATGCATT
CAACATCGCCAGATATCAATTAGGCATAGAGAAACTACTCGATCCTGAAGATGT
TGATACCACCTATCCAGATAAGAAGTCCATCTTAATGTACATCACATCACTCTTC
CAAGTTTTGCCTCAACAAGTGAGCATTGAAGCCATCCAGGAAGTGGAAATGTTG
CCAAGGCCACCTAAAGTGACTAAAGAAGAACATTTTCAGTTACATCATCAAATG
CACTATTCTCAACAGATCACGGTCAGTCTAGCACAGGGATATGAGAGAACTTCTT
CCCCTAAGCCTCGATTCAAGAGCTATGCCTACACACAGGCTGCTTATGTCACCAC
CTCTGACCCTACACGGAGCCCATTTCCTTCACAGCATTTGGAAGCTCCTGAAGAC

AAGTCATTTGGCAGTTCATTGATGGAGAGTGAAGTAAACCTGGACCGTTATCAAA
CAGCTTTAGAAGAAGTATTATCGTGGCTTCTTTCTGCTGAGGACACATTGCAAGC
ACAAGGAGAGATTTCTAATGATGTGGAAGTGGTGAAAGACCAGTTTCATACTCA
TGAGGGGTACATGATGGATTTGACAGCCCATCAGGGCCGGGTTGGTAATATTCTA
CAATTGGGAAGTAAGCTGATTGGAACAGGAAAATTATCAGAAGATGAAGAAACT
GAAGTACAAGAGCAGATGAATCTCCTAAATTCAAGATGGGAATGCCTCAGGGTA
GCTAGCATGGAAAAACAAAGCAATTTACATAGAGTTTTAATGGATCTCCAGAAT
CAGAAACTGAAAGAGTTGAATGACTGGCTAACAAAAACAGAAGAAAGAACAAG
GAAAATGGAGGAAGAGCCTCTTGGACCTGATCTTGAAGACCTAAAACGCCAAGT
ACAACAACATAAGGTGCTTCAAGAAGATCTAGAACAAGAACAAGTCAGGGTCAA
TTCTCTCACTCACATGGTGGTGGTAGTTGATGAATCTAGTGGAGATCACGCAACT
GCTGCTTTGGAAGAACAACTTAAGGTATTGGGAGATCGATTTGCAGCCATTTCAC
ACAGAATTAAGACTGGAAAGGCCTCCATTCCTTTGAAGGAATTGGAGCAGTTTA
ACTCAGATATACAAAAATTGCTTGAACCACTGGAGGCTGAAATTCAGCAGGGGG
TGAATCTGAAAGAGGAAGACTTCAATAAAGATATGAATGAAGACAATGAGGGTA
CTGTAAAAGAATTGTTGCAAAGAGGAGACAACTTACAACAAAGAATCACAGATG
AGAGAAAGCGAGAGGAAATAAAGATAAAACAGCAGCTGTTACAGACAAAACAT
AATGCTCTCAAGGATTTGAGGTCTCAAAGAAGAAAAAAGGCTCTAGAAATTTCT
CATCAGTGGTATCAGTACAAGAGGCAGGCTGATGATCTCCTGAAATGCTTGGAT
GACATTGAAAAAAAATTAGCCAGCCTACCTGAGCCCAGAGATGAAAGGAAAATA
AAGGAAATTGATCGGGAATTGCAGAAGAAGAAAGAGGAGCTGAATGCAGTGCG
TAGGCAAGCTGAGGGCTTGTCTGAGGATGGGGCCGCAATGGCAGTGGAGCCAAC
TCAGATCCAGCTCAGCAAGCGCTGGCGGGAAATTGAGAGCAAATTTGCTCAGTT
TCGAAGACTCAACTTTGCACAAATTCACACTGTCCGTGAAGAAACGATGATGGT
GATGACTGAAGACATGCCTTTGGAAATTTCTTATGTGCCTTCTACTTATTTGACTG

AAATCACTCATGTCTCACAAGCCCTATTAGAAGTGGAACAACTTCTCAATGCTCC
TGACCTCTGTGCTAAGGACTTTGAAGATCTCTTTAAGCAAGAGGAGTCTCTGAAG
AATATAAAAGATAGTCTACAACAAAGCTCAGGTCGGATTGACATTATTCATAGC
AAGAAGACAGCAGCATTGCAAAGTGCAACGCCTGTGGAAAGGGTGAAGCTACA
GGAAGCTCTCTCCCAGCTTGATTTCCAATGGGAAAAAGTTAACAAAATGTACAA
GGACCGACAAGGGCGATTTGACAGATCTGTTGAGAAATGGCGGCGTTTTCATTAT
GATATAAAGATATTTAATCAGTGGCTAACAGAAGCTGAACAGTTTCTCAGAAAG
ACACAAATTCCTGAGAATTGGGAACATGCTAAATACAAATGGTATCTTAAGGAA
CTCCAGGATGGCATTGGGCAGCGGCAAACTGTTGTCAGAACATTGAATGCAACT
GGGGAAGAAATAATTCAGCAATCCTCAAAAACAGATGCCAGTATTCTACAGGAA
AAATTGGGAAGCCTGAATCTGCGGTGGCAGGAGGTCTGCAAACAGCTGTCAGAC
AGAAAAAAGAGGCTAGAAGAACAAAAGAATATCTTGTCAGAATTTCAAAGAGAT
TTAAATGAATTTGTTTTATGGTTGGAGGAAGCAGATAACATTGCTAGTATCCCAC
TTGAACCTGGAAAAGAGCAGCAACTAAAAGAAAAGCTTGAGCAAGTCAAGTTAC
TGGTGGAAGAGTTGCCCCTGCGCCAGGGCCGAATTCTCAAACAATTAAATGAAA
CTGGAGGACCCGTGCTTGTAAGTGCTCCCATAAGCCCAGAAGAGCAAGATAAAC
TTGAAAATAAGCTCAAGCAGACAAATCTCCAGTGGATAAAGGTTTCCAGAGCTT
TACCTGAGAAACAAGGAGAAATTGAAGCTCAAATAAAAGACCTTGGGCAGCTTG
AAAAAAAGCTTGAAGACCTTGAAGAGCAGTTAAATCATCTGCTGCTGTGGTTATC
TCCTATTAGGAATCAGTTGGAAATTTATAACCAACCAAACCAAGAAGGACCATTT
GACGTTAAGGAAACTGAAATAGCAGTTCAAGCTAAACAACCGGATGTGGAAGAG
ATTTTGTCTAAAGGGCAGCATTTGTACAAGGAAAAACCAGCCACTCAGCCAGTG
AAGAGGAAGTTAGAAGATCTGAGCTCTGAGTGGAAGGCGGTAAACCGTTTACTT
CAAGAGCTGAGGGCAAAGCAGCCTGACCTAGCTCCTGGACTGACCACTATTGGA
GCCTCTCCTACTCAGACTGTTACTCTGGTGACACAACCTGTGGTTACTAAGGAAA

CTGCCATCTCCAAACTAGAAATGCCATCTTCCTTGATGTTGGAGGTACCTGCTCT

GGCAGATTTCAACCGGGCTTGGACAGAACTTACCGACTGGCTTTCTCTGCTTGAT

CAAGTTATAAAATCACAGAGGGTGATGGTGGGTGACCTTGAGGATATCAACGAG

ATGATCATCAAGCAGAAGGCAACAATGCAGGATTTGGAACAGAGGCGTCCCCAG

TTGGAAGAACTCATTACCGCTGCCCAAAATTTGAAAAACAAGACCAGCAATCAA

GAGGCTAGAACAATCATTACGGATCGAATTGAAAGAATTCAGAATCAGTGGGAT

GAAGTACAAGAACACCTTCAGAACCGGAGGCAACAGTTGAATGAAATGTTAAAG

GATTCAACACAATGGCTGGAAGCTAAGGAAGAAGCTGAGCAGGTCTTAGGACAG

GCCAGAGCCAAGCTTGAGTCATGGAAGGAGGGTCCCTATACAGTAGATGCAATC

CAAAAGAAAATCACAGAAACCAAGCAGTTGGCCAAAGACCTCCGCCAGTGGCA

GACAAATGTAGATGTGGCAAATGACTTGGCCCTGAAACTTCTCCGGGATTATTCT

GCAGATGATACCAGAAAAGTCCACATGATAACAGAGAATATCAATGCCTCTTGG

AGAAGCATTCATAAAAGGGTGAGTGAGCGAGAGGCTGCTTTGGAAGAAACTCAT

AGATTACTGCAACAGTTCCCCCTGGACCTGGAAAAGTTTCTTGCCTGGCTTACAG

AAGCTGAAACAACTGCCAATGTCCTACAGGATGCTACCCGTAAGGAAAGGCTCC

TAGAAGACTCCAAGGGAGTAAAAGAGCTGATGAAACAATGGCAAGACCTCCAA

GGTGAAATTGAAGCTCACACAGATGTTTATCACAACCTGGATGAAAACAGCCAA

AAAATCCTGAGATCCCTGGAAGGTTCCGATGATGCAGTCCTGTTACAAAGACGTT

TGGATAACATGAACTTCAAGTGGAGTGAACTTCGGAAAAAGTCTCTCAACATTA

GGTCCCATTTGGAAGCCAGTTCTGACCAGTGGAAGCGTCTGCACCTTTCTCTGCA

GGAACTTCTGGTGTGGCTACAGCTGAAAGATGATGAATTAAGCCGGCAGGCACC

TATTGGAGGCGACTTTCCAGCAGTTCAGAAGCAGAACGATGTACATAGGGCCTT

CAAGAGGGAATTGAAAACTAAAGAACCTGTAATCATGAGTACTCTTGAGACTGT

ACGAATATTTCTGACAGAGCAGCCTTTGGAAGGACTAGAGAAACTCTACCAGGA

GCCCAGAGAGCTGCCTCCTGAGGAGAGAGCCCAGAATGTCACTCGGCTTCTACG

AAAGCAGGCTGAGGAGGTCAATACTGAGTGGAAAAATTGAACCTGCACTCCGC

TGACTGGCAGAGAAAAATAGATGAGACCCTTGAAAGACTCCAGGAACTTCAAGA

GGCCACGGATGAGCTGGACCTCAAGCTGCGCCAAGCTGAGGTGATCAAGGGATC

CTGGCAGCCCGTGGGCGATCTCCTCATTGACTCTCTCCAAGATCACCTCGAGAAA

GTCAAGGCACTTCGAGGAGAAATTGCGCCTCTGAAAGAGAACGTGAGCCACGTC

AATGACCTTGCTCGCCAGCTTACCACTTTGGGCATTCAGCTCTCACCGTATAACC

TCAGCACTCTGGAAGACCTGAACACCAGATGGAAGCTTCTGCAGGTGGCCGTCG

AGGACCGAGTCAGGCAGCTGCATGAAGCCCACAGGGACTTTGGTCCAGCATCTC

AGCACTTTCTTTCCACGTCTGTCCAGGGTCCCTGGGAGAGAGCCATCTCGCCAAA

CAAAGTGCCCTACTATATCAACCACGAGACTCAAACAACTTGCTGGGACCATCCC

AAAATGACAGAGCTCTACCAGTCTTTAGCTGACCTGAATAATGTCAGATTCTCAG

CTTATAGGACTGCCATGAAACTCCGAAGACTGCAGAAGGCCCTTTGCTTGGATCT

CTTGAGCCTGTCAGCTGCATGTGATGCCTTGGACCAGCACAACCTCAAGCAAAAT

GACCAGCCCATGGATATCCTGCAGATTATTAATTGTTTGACCACTATTTATGACC

GCCTGGAGCAAGAGCACAACAATTTGGTCAACGTCCCTCTCTGCGTGGATATGTG

TCTGAACTGGCTGCTGAATGTTTATGATACGGGACGAACAGGGAGGATCCGTGT

CCTGTCTTTTAAAACTGGCATCATTTCCCTGTGTAAAGCACATTTGGAAGACAAG

TACAGATACCTTTTCAAGCAAGTGGCAAGTTCAACAGGATTTTGTGACCAGCGCA

GGCTGGGCCTCCTTCTGCATGATTCTATCCAAATTCCAAGACAGTTGGGTGAAGT

TGCATCCTTTGGGGGCAGTAACATTGAGCCAAGTGTCCGGAGCTGCTTCCAATTT

GCTAATAATAAGCCAGAGATCGAAGCGGCCCTCTTCCTAGACTGGATGAGACTG

GAACCCCAGTCCATGGTGTGGCTGCCCGTCCTGCACAGAGTGGCTGCTGCAGAA

ACTGCCAAGCATCAGGCCAAATGTAACATCTGCAAAGAGTGTCCAATCATTGGA

TTCAGGTACAGGAGTCTAAAGCACTTTAATTATGACATCTGCCAAAGCTGCTTTT

TTTCTGGTCGAGTTGCAAAAGGCCATAAAATGCACTATCCCATGGTGGAATATTG

CACTCCGACTACATCAGGAGAAGATGTTCGAGACTTTGCCAAGGTACTAAAAAA

CAAATTTCGAACCAAAAGGTATTTTGCGAAGCATCCCCGAATGGGCTACCTGCCA

GTGCAGACTGTCTTAGAGGGGGACAACATGGAAACTCCCGTTACTCTGATCAACT

TCTGGCCAGTAGATTCTGCGCCTGCCTCGTCCCCTCAGCTTTCACACGATGATACT

CATTCACGCATTGAACATTATGCTAGCAGGCTAGCAGAAATGGAAAACAGCAAT

GGATCTTATCTAAATGATAGCATCTCTCCTAATGAGAGCATAGATGATGAACATT

TGTTAATCCAGCATTACTGCCAAAGTTTGAACCAGGACTCCCCCCTGAGCCAGCC

TCGTAGTCCTGCCCAGATCTTGATTTCCTTAGAGAGTGAGGAAAGAGGGGAGCT

AGAGAGAATCCTAGCAGATCTTGAGGAAGAAAACAGGAATCTGCAAGCAGAAT

ATGACCGTCTAAAGCAGCAGCACGAACATAAAGGCCTGTCCCCACTGCCGTCCC

CTCCTGAAATGATGCCCACCTCTCCCCAGAGTCCCCGGGATGCTGAGCTCATTGC

TGAGGCCAAGCTACTGCGTCAACACAAAGGCCGCCTGGAAGCCAGGATGCAAAT

CCTGGAAGACCACAATAAACAGCTGGAGTCACAGTTACACAGGCTAAGGCAGCT

GCTGGAGCAACCCCAGGCAGAGGCCAAAGTGAATGGCACAACGGTGTCCTCTCC

TTCTACCTCTCTACAGAGGTCCGACAGCAGTCAGCCTATGCTGCTCCGAGTGGTT

GGCAGTCAAACTTCGGACTCCATGGGTGAGGAAGATCTTCTCAGTCCTCCCCAGG

ACACAAGCACAGGGTTAGAGGAGGTGATGGAGCAACTCAACAACTCCTTCCCTA

GTTCAAGAGGAAGAAATACCCCTGGAAAGCCAATGAGAGAGGACACAATGGCC

ACAACCATGGTGAGCAAGGGCGAGGAGCTGTTCACCGGGGTGGTGCCCATCCTG

GTCGAGCTGGACGGCGACGTAAACGGCCACAAGTTCAGCGTGTCCGGCGAGGGC

GAGGGCGATGCCACCTACGGCAAGCTGACCCTGAAGTTCATCTGCACCACCGGC

AAGCTGCCCGTGCCCTGGCCCACCCTCGTGACCACCCTGACCTACGGCGTGCAGT

GCTTCAGCCGCTACCCCGACCACATGAAGCAGCACGACTTCTTCAAGTCCGCCAT

GCCCGAAGGCTACGTCCAGGAGCGCACCATCTTCTTCAAGGACGACGGCAACTA

CAAGACCCGCGCCGAGGTGAAGTTCGAGGGCGACACCCTGGTGAACCGCATCGA

GCTGAAGGGCATCGACTTCAAGGAGGACGGCAACATCCTGGGGCACAAGCTGGA
GTACAACTACAACAGCCACAACGTCTATATCATGGCCGACAAGCAGAAGAACGG
CATCAAGGTGAACTTCAAGATCCGCCACAACATCGAGGACGGCAGCGTGCAGCT
CGCCGACCACTACCAGCAGAACACCCCCATCGGCGACGGCCCCGTGCTGCTGCC
CGACAACCACTACCTGAGCACCCAGTCCGCCCTGAGCAAAGACCCCAACGAGAA
GCGCGATCACATGGTCCTGCTGGAGTTCGTGACCGCCGCCGGGATCACTCTCGGC
ATGGACGAGCTGTACAAGTAAA

**Sequence of C2 construct (from ATG to EGFP):**

ATGCTTTGGTGGGAAGAAGTAGAGGACTGTTATGAAAGAGAAGATGTTCAAAAG
AAAACATTCACAAAATGGGTAAATGCACAATTTTCTAAGTTTGGGAAGCAGCAT
ATTGAGAACCTCTTCAGTGACCTACAGGATGGGAGGCGCCTCCTAGACCTCCTCG
AAGGCCTGACAGGGCAAAAACTGCCAAAAGAAAAAGGATCCACAAGAGTTCAT
GCCCTGAACAATGTCAACAAGGCACTGCGGGTTTTGCAGAACAATAATGTTGATT
TAGTGAATATTGGAAGTACTGACATCGTAGATGGAAATCATAAACTGACTCTTGG
TTTGATTTGGAATATAATCCTCCACTGGCAGGTCAAAAATGTAATGAAAAATATC
ATGGCTGGATTGCAACAAACCAACAGTGAAAAGATTCTCCTGAGCTGGGTCCGA
CAATCAACTCGTAATTATCCACAGGTTAATGTAATCAACTTCACCACCAGCTGGT
CTGATGGCCTGGCTTTGAATGCTCTCATCCATAGTCATAGGCCAGACCTATTTGA
CTGGAATAGTGTGGTTTGCCAGCAGTCAGCCACACAACGACTGGAACATGCATT
CAACATCGCCAGATATCAATTAGGCATAGAGAAACTACTCGATCCTGAAGATGT
TGATACCACCTATCCAGATAAGAAGTCCATCTTAATGTACATCACATCACTCTTC
CAAGTTTTGCCTCAACAAGTGAGCATTGAAGCCATCCAGGAAGTGGAAATGTTG
CCAAGGCCACCTAAAGTGACTAAAGAAGAACATTTTCAGTTACATCATCAAATG
CACTATTCTCAACAGATCACGGTCAGTCTAGCACAGGGATATGAGAGAACTTCTT

CCCCTAAGCCTCGATTCAAGAGCTATGCCTACACACAGGCTGCTTATGTCACCAC

CTCTGACCCTACACGGAGCCCATTTCCTTCACAGCATTTGGAAGCTCCTGAAGAC

AAGTCATTTGGCAGTTCATTGATGGAGAGTGAAGTAAACCTGGACCGTTATCAAA

CAGCTTTAGAAGAAGTATTATCGTGGCTTCTTTCTGCTGAGGACACATTGCAAGC

ACAAGGAGAGATTTCTAATGATGTGGAAGTGGTGAAAGACCAGTTTCATACTCA

TGAGGGGTACATGATGGATTTGACAGCCCATCAGGGCCGGGTTGGTAATATTCTA

CAATTGGGAAGTAAGCTGATTGGAACAGGAAAATTATCAGAAGATGAAGAAACT

GAAGTACAAGAGCAGATGAATCTCCTAAATTCAAGATGGGAATGCCTCAGGGTA

GCTAGCATGGAAAAACAAAGCAATTTACATAGAGTTTTAATGGATCTCCAGAAT

CAGAAACTGAAAGAGTTGAATGACTGGCTAACAAAAACAGAAGAAAGAACAAG

GAAAATGGAGGAAGAGCCTCTTGGACCTGATCTTGAAGACCTAAAACGCCAAGT

ACAACAACATAAGGTGCTTCAAGAAGATCTAGAACAAGAACAAGTCAGGGTCAA

TTCTCTCACTCACATGGTGGTGGTAGTTGATGAATCTAGTGGAGATCACGCAACT

GCTGCTTTGGAAGAACAACTTAAGGTATTGGGAGATCGATGGGCAAACATCTGT

AGATGGACAGAAGACCGCTGGGTTCTTTTACAAGACATCCTTCTCAAATGGCAAC

GTCTTACTGAAGAACAGTGCCTTTTTAGTGCATGGCTTTCAGAAAAAGAAGATGC

AGTGAACAAGATTCACACAACTGGCTTTAAAGATCAAAATGAAATGTTATCAAG

TCTTCAAAAACTGGCCGTTTTAAAAGCGGATCTAGAAAAGAAAAAGCAATCCAT

GGGCAAACTGTATTCACTCAAACAAGATCTTCTTTCAACACTGAAGAATAAGTCA

GTGACCCAGAAGACGGAAGCATGGCTGGATAACTTTGCCCGGTGTTGGGATAAT

TTAGTCCAAAAACTTGAAAAGAGTACAGCACAGACCCTTGAAAGACTCCAGGAA

CTTCAAGAGGCCATGGATGAGCTGGACCTCAAGCTGCGCCAAGCTGAGGTGATC

AAGGGATCCTGGCAGCCCGTGGGCGATCTCCTCATTGACTCTCTCCAAGATCACC

TCGAGAAAGTCAAGGCACTTCGAGGAGAAATTGCGCCTCTGAAAGAGAACGTGA

GCCACGTCAATGACCTTGCTCGCCAGCTTACCACTTTGGGCATTCAGCTCTCACC

GTATAACCTCAGCACTCTGGAAGACCTGAACACCAGATGGAAGCTTCTGCAGGT

GGCCGTCGAGGACCGAGTCAGGCAGCTGCATGAAGCCCACAGGGACTTTGGTCC

AGCATCTCAGCACTTTCTTTCCACGTCTGTCCAGGGTCCCTGGGAGAGAGCCATC

TCGCCAAACAAAGTGCCCTACTATATCAACCACGAGACTCAAACAACTTGCTGG

GACCATCCCAAAATGACAGAGCTCTACCAGTCTTTAGCTGACCTGAATAATGTCA

GATTCTCAGCTTATAGGACTGCCATGAAACTCCGAAGACTGCAGAAGGCCCTTTG

CTTGGATCTCTTGAGCCTGTCAGCTGCATGTGATGCCTTGGACCAGCACAACCTC

AAGCAAAATGACCAGCCCATGGATATCCTGCAGATTATTAATTGTTTGACCACTA

TTTATGACCGCCTGGAGCAAGAGCACAACAATTTGGTCAACGTCCCTCTCTGCGT

GGATATGTGTCTGAACTGGCTGCTGAATGTTTATGATACGGGACGAACAGGGAG

GATCCGTGTCCTGTCTTTTAAAACTGGCATCATTTCCCTGTGTAAAGCACATTTGG

AAGACAAGTACAGATACCTTTTCAAGCAAGTGGCAAGTTCAACAGGATTTTGTG

ACCAGCGCAGGCTGGGCCTCCTTCTGCATGATTCTATCCAAATTCCAAGACAGTT

GGGTGAAGTTGCATCCTTTGGGGGCAGTAACATTGAGCCAAGTGTCCGGAGCTG

CTTCCAATTTGCTAATAATAAGCCAGAGATCGAAGCGGCCCTCTTCCTAGACTGG

ATGAGACTGGAACCCCAGTCCATGGTGTGGCTGCCCGTCCTGCACAGAGTGGCT

GCTGCAGAAACTGCCAAGCATCAGGCCAAATGTAACATCTGCAAAGAGTGTCCA

ATCATTGGATTCAGGTACAGGAGTCTAAAGCACTTTAATTATGACATCTGCCAAA

GCTGCTTTTTTTCTGGTCGAGTTGCAAAAGGCCATAAAATGCACTATCCCATGGT

GGAATATTGCACTCCGACTACATCAGGAGAAGATGTTCGAGACTTTGCCAAGGT

ACTAAAAAACAAATTTCGAACCAAAAGGTATTTTGCGAAGCATCCCCGAATGGG

CTACCTGCCAGTGCAGACTGTCTTAGAGGGGGACAACATGGAAACTCCCGTTACT

CTGATCAACTTCTGGCCAGTAGATTCTGCGCCTGCCTCGTCCCCTCAGCTTTCACA

CGATGATACTCATTCACGCATTGAACATTATGCTAGCAGGCTAGCAGAAATGGA

AAACAGCAATGGATCTTATCTAAATGATAGCATCTCTCCTAATGAGAGCATAGAT

GATGAACATTTGTTAATCCAGCATTACTGCCAAAGTTTGAACCAGGACTCCCCCC

TGAGCCAGCCTCGTAGTCCTGCCCAGATCTTGATTTCCTTAGAGAGTGAGGAAAG

AGGGGAGCTAGAGAGAATCCTAGCAGATCTTGAGGAAGAAAACAGGAATCTGC

AAGCAGAATATGACCGTCTAAAGCAGCAGCACGAACATAAAGGCCTGTCCCCAC

TGCCGTCCCCTCCTGAAATGATGCCCACCTCTCCCCAGAGTCCCCGGGATGCTGA

GCTCATTGCTGAGGCCAAGCTACTGCGTCAACACAAAGGCCGCCTGGAAGCCAG

GATGCAAATCCTGGAAGACCACAATAAACAGCTGGAGTCACAGTTACACAGGCT

AAGGCAGCTGCTGGAGCAACCCCAGGCAGAGGCCAAAGTGAATGGCACAACGG

TGTCCTCTCCTTCTACCTCTCTACAGAGGTCCGACAGCAGTCAGCCTATGCTGCTC

CGAGTGGTTGGCAGTCAAACTTCGGACTCCATGGGTGAGGAAGATCTTCTCAGTC

CTCCCCAGGACACAAGCACAGGGTTAGAGGAGGTGATGGAGCAACTCAACAACT

CCTTCCCTAGTTCAAGAGGAAGAAATACCCCTGGAAAGCCAATGAGAGAGGACA

CAATGGCCACAACCATGGTGAGCAAGGGCGAGGAGCTGTTCACCGGGGTGGTGC

CCATCCTGGTCGAGCTGGACGGCGACGTAAACGGCCACAAGTTCAGCGTGTCCG

GCGAGGGCGAGGGCGATGCCACCTACGGCAAGCTGACCCTGAAGTTCATCTGCA

CCACCGGCAAGCTGCCCGTGCCCTGGCCCACCCTCGTGACCACCCTGACCTACGG

CGTGCAGTGCTTCAGCCGCTACCCCGACCACATGAAGCAGCACGACTTCTTCAAG

TCCGCCATGCCCGAAGGCTACGTCCAGGAGCGCACCATCTTCTTCAAGGACGAC

GGCAACTACAAGACCCGCGCCGAGGTGAAGTTCGAGGGCGACACCCTGGTGAAC

CGCATCGAGCTGAAGGGCATCGACTTCAAGGAGGACGGCAACATCCTGGGGCAC

AAGCTGGAGTACAACTACAACAGCCACAACGTCTATATCATGGCCGACAAGCAG

AAGAACGGCATCAAGGTGAACTTCAAGATCCGCCACAACATCGAGGACGGCAGC

GTGCAGCTCGCCGACCACTACCAGCAGAACACCCCCATCGGCGACGGCCCCGTG

CTGCTGCCCGACAACCACTACCTGAGCACCCAGTCCGCCCTGAGCAAAGACCCC

AACGAGAAGCGCGATCACATGGTCCTGCTGGAGTTCGTGACCGCCGCCGGGATC ACTCTCGGCATGGACGAGCTGTACAAGTAAA

**Table S1. Primers spanning the promoter to EGFP region of the constructs used for PCR on genomic DNAs extracted from SFFV-C1-GFP and SFFV-C2-GFP transduced cells.**

| Cell type | PCR fragment | Forward primer | Reverse primer | Size |
|---|---|---|---|---|
| SC1 | 1 | ACCCATCAGATGTTTCCAGGC | ATGCATGTTCCAGTCGTTGTG | 842bp |
| | 2 | GAGCTGGGTCCGACAATCAA | CATGCTAGCTACCCTGAGGC | 894bp |
| | 3 | TGGGAAGTAAGCTGATTGGAACA | CTTGCCTACGCACTGCATTC | 920bp |
| | 4 | GTACAAGAGGCAGGCTGATGA | AATGCCATCCTGGAGTTCCTT | 813bp |
| | 5 | CGACAAGGGCGATTTGACAG | CCTCTTCACTGGCTGAGTGG | 923bp |
| | 6 | AGCAGTTCAAGCTAAACAACCG | TGACTCAAGCTTGGCTCTGG | 705bp |
| | 7 | AACCGGAGGCAACAGTTGAA | GACGCTTCCACTGGTCAGAA | 720bp |
| | 8 | TCCTGAGATCCCTGGAAGGTT | GACGTGGCTCACGTTCTCTT | 700bp |
| | 9 | TCAAGAGGCCACGGATGAG | TATCCACGCAGAGAGGGACG | 712bp |
| | 10 | CCTTGGACCAGCACAACCTC | AGAGTAACGGGAGTTTCCATGT | 840bp |
| | 11 | TGCACTCCGACTACATCAGG | TGGTTGTGGCCATTGTGTCC | 987bp |
| | 12 | AGCACAGGGTTAGAGGAGGT | CTCAGGTAGTGGTTGTCGGG | 715bp |
| SC2 | 1 | ACCCATCAGATGTTTCCAGGC | ATGCATGTTCCAGTCGTTGTG | 842bp |
| | 2 | GAGCTGGGTCCGACAATCAA | CATGCTAGCTACCCTGAGGC | 894bp |
| | 3 | TGGGAAGTAAGCTGATTGGAACA | TACGGTGAGAGCTGAATGCC | 1030bp |
| | 4 | AGAAAAAGCAATCCATGGGCA | TATCCACGCAGAGAGGGACG | 894bp |
| | 5 | CCTTGGACCAGCACAACCTC | AGATCTGGGCAGGACTACGA | 1089bp |
| | 6 | CCCTCAGCTTTCACACGATGA | CTCAGGTAGTGGTTGTCGGG | 1398bp |
| | 7 | AGCACAGGGTTAGAGGAGGT | CTCAGGTAGTGGTTGTCGGG | 715bp |

**Supplementary Figure legends:**

Figure S1.Transduction efficiency of SFFV-C1-GFP, hDesmin-C1-GFP, SFFV-C2-GFP, hDesmin-C2-GFP, SFFV-GFP and hDesmin-GFP lentivirus in DMD pericytes, analysed by FACS.

Figure S2: Full length, uncropped western blotting image of Figure 2B showing the expression of GFP (red) and tubulin 2.1 (green) by proliferating CD133+ cells (negative

control), non-transduced pericytes (NT), pericytes transduced with SFFV-GFP (SG), hDesmin-GFP (DG), SFFV-C1-GFP (SC1), hDesmin-C1-GFP (DC1), SFFV-C2-GFP (SC2) and hDesmin-C2-GFP(DC2) lentiviruses. GFP bands of 27KD were expressed by SG and DG cells. A GFP band of 312KD was expressed by SC1 cells, and a 168KD GFP band by SC2 and DC2 cells; all of these are the expected molecular weight.

Figure S3: Detection of dystrophin C1-GFP in cells transduced with SFFV-C1-GFP (SC1) or Desmin-C1-GFP (DC1) lentiviruses, by western blot using either dystrophin or GFP antibodies. Mouse anti-GAPDH antibody was used as loading control. Cells maintained *in vitro* under both proliferating and differentiated conditions were analysed. Both dystrophin and GFP antibodies recognize the same protein at the expected molecular weight.

Figure S4: Full length, uncropped western blotting image of Figure S3 showing 2 western blots that were run in parallel, to determine the staining pattern of poly-dystrophin antibody and GFP antibody on cells transduced with SC1 or DC1 lentiviruses. Blot 1 (A, B, and C) was incubated with polyclonal dystrophin antibody (A and C, red) and the GAPDH antibody (B and C, green), blot 2 was incubated with polyclonal GFP (D and F, red), myosin (MF20, green in E and F, 200KD band) and GAPDH antibody (green in E and F, 37KD). C and F are merged image of A, B and D, E, respectively. The results show similar staining pattern of dystrophin and GFP antibody by western blot, thus GFP was subsequently used as surrogate marker for dystrophin in this study. Sample ID: Lane 1: none transduced cells; 2: SC1 transduced cells (non-differentiated); 3: DC1 transduced cells (non-differentiated); 4: SC1 transduced cells 7 days after differentiation; 5: DC1 transduced cells 7 days after differentiation.

Figure S1

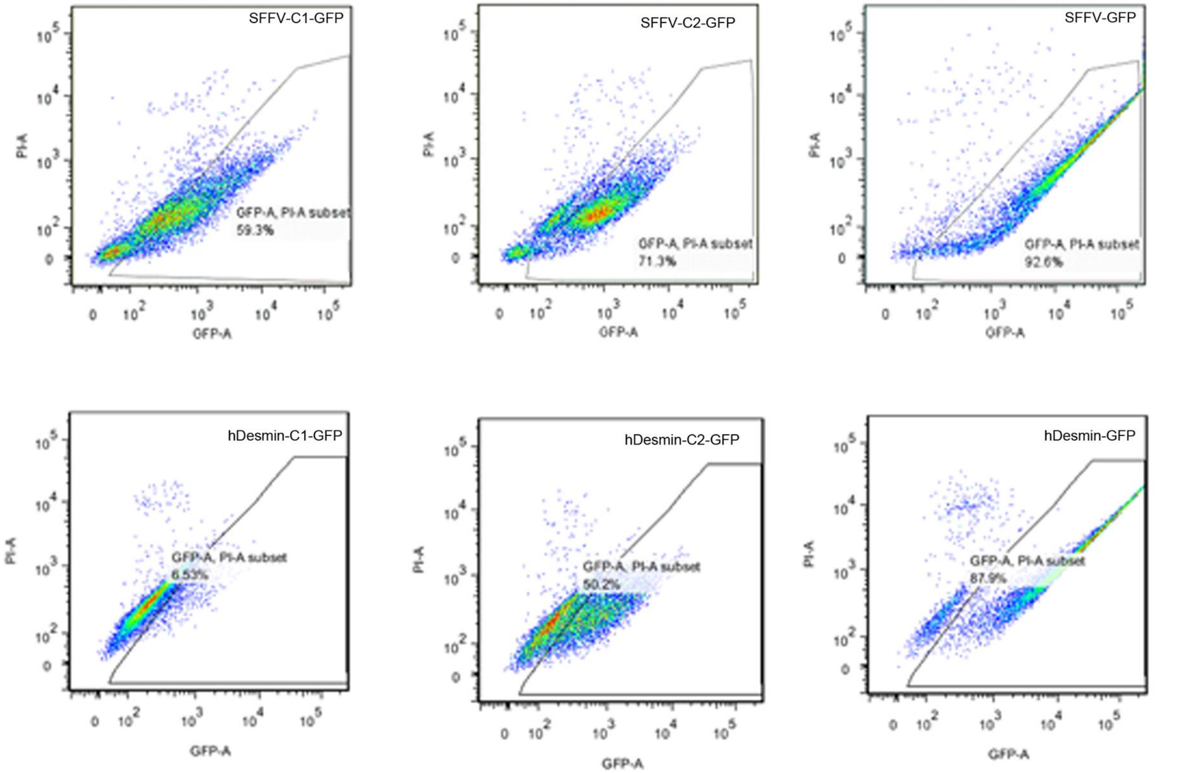
SFFV-C1-GFP
GFP-A, PI-A subset
59.3%
SFFV-C2-GFP
GFP-A, PI-A subset
71.3%
SFFV-GFP
GFP-A, PI-A subset
92.6%
hDesmin-C1-GFP
GFP-A, PI-A subset
6.53%
hDesmin-C2-GFP
GFP-A, PI-A subset
50.2%
hDesmin-GFP
GFP-A, PI-A subset
87.9%
PI-A
GFP-A

Figure S2

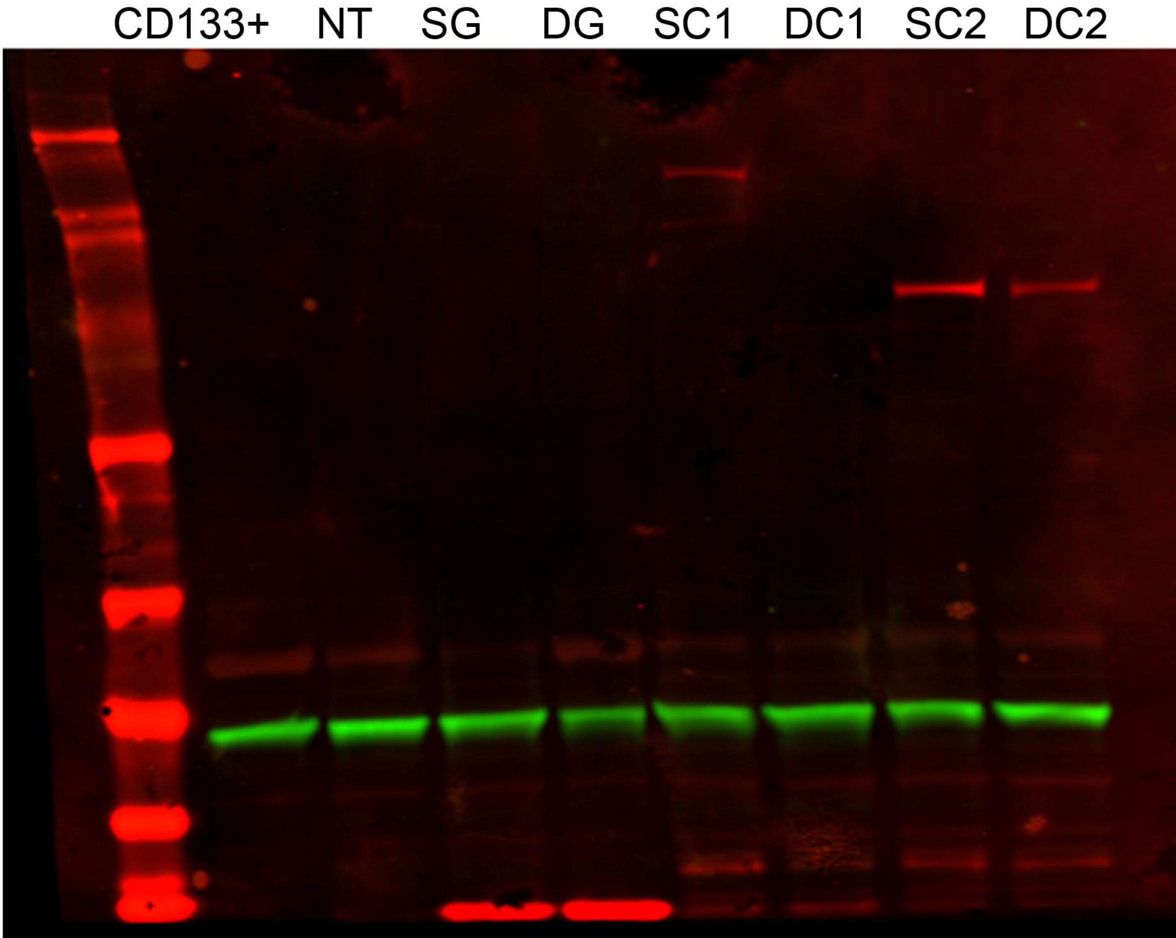

Figure S3

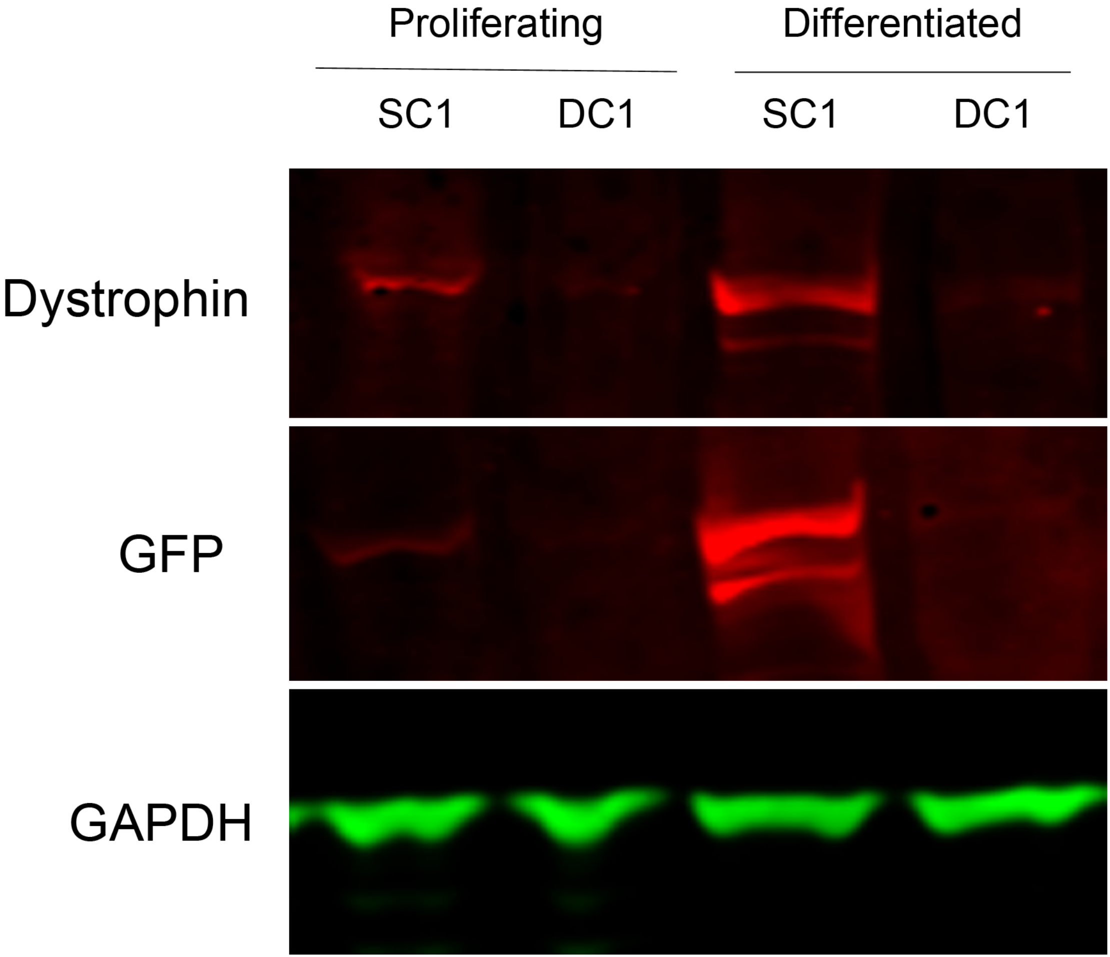
Proliferating
Differentiated
SC1
DC1
SC1
DC1
Dystrophin
GFP
GAPDH

Figure S4

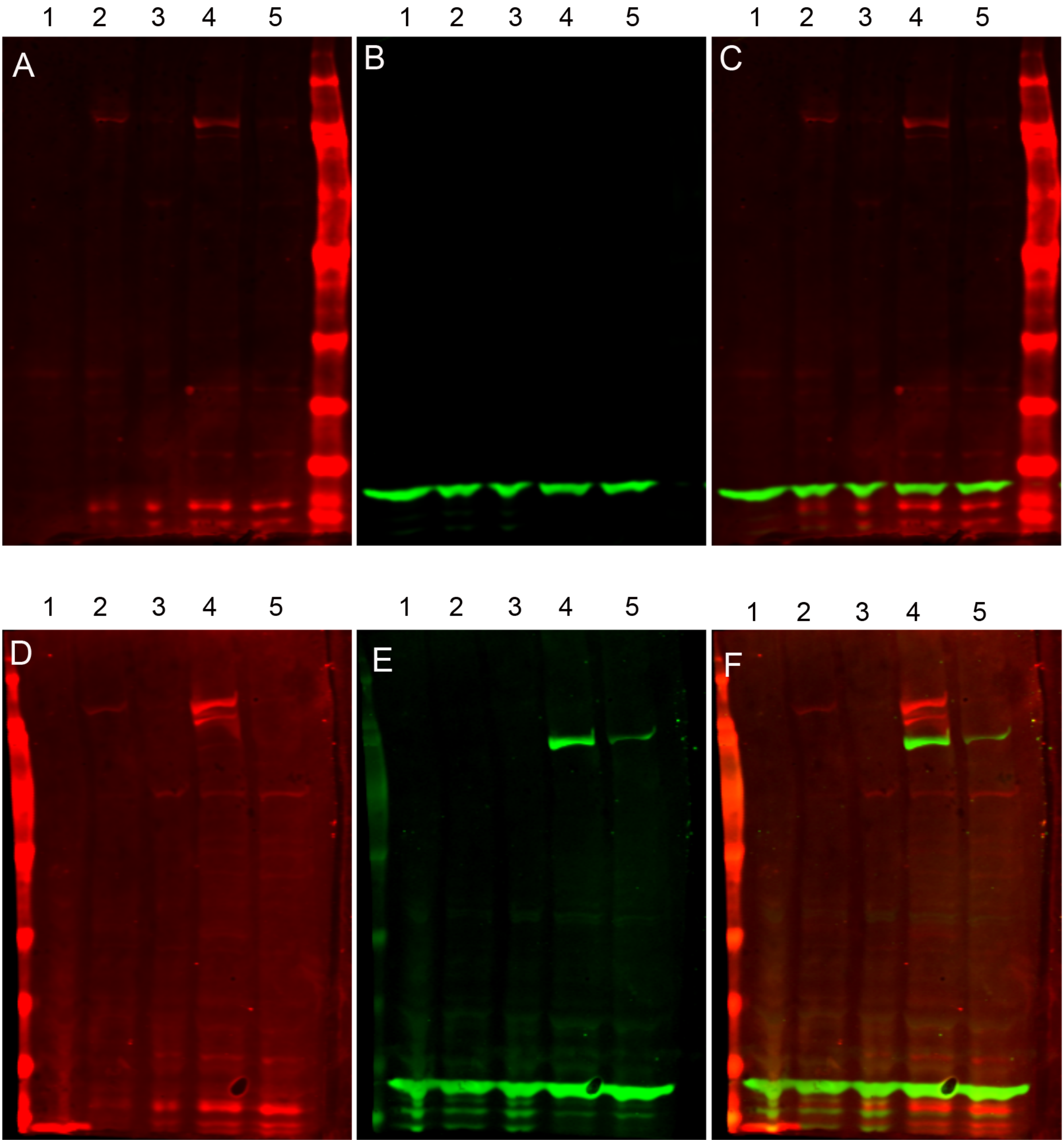

Supplement: Supplementary Information [file srep19750-s1.pdf]
